# CircRNA hsa_circ_0004781 promoted cell proliferation by acting as a sponge for miR-9-5p and miR-338-3p and upregulating *KLF5* and *ADAM17* expression in pancreatic ductal adenocarcinoma

**DOI:** 10.1186/s12935-025-03687-0

**Published:** 2025-02-19

**Authors:** Kun-Lin Lee, Jun-Jen Liu, Wei-Jan Huang, Ching-Sheng Hung, Yu-Chih Liang

**Affiliations:** 1 Ph.D. Program in Medical Biotechnology, College of Medical Science and Technology, Taipei Medical University, New Taipei City, Taiwan; 2 School of Medical Laboratory Science and Biotechnology, College of Medical Science and Technology, Taipei Medical University, 301 Yuantong Rd., New Taipei City, 23564 Taiwan; 3 School of Pharmacy, College of Pharmacy, Taipei Medical University, Taipei, Taiwan; 4 Ph.D. Program in Drug Discovery and Development Industry, College of Pharmacy, Taipei Medical University, Taipei, Taiwan; 5 Department of Laboratory Medicine, Wan Fang Hospital, Taipei Medical University, Taipei, Taiwan; 6 Traditional Herbal Medicine Research Center, Taipei Medical University Hospital, Taipei, Taiwan

**Keywords:** Real circRNA, Hsa_circ_0004781, miR-9-5p, miR-338-3p, KLF5, ADAM17, Pancreatic ductal adenocarcinoma

## Abstract

**Background:**

Pancreatic ductal adenocarcinoma (PDAC) is one of the most aggressive types of solid tumor, and novel strategies must be developed for treating it. Previous studies predominantly utilized circular RNA (circRNA) expression plasmids incorporating Alu elements to facilitate the indirect expression of circRNA.

**Methods:**

Public databases and bioinformatics tools were used to identify hsa_circ_0004781 that is highly expressed in PDAC and its potential microRNA (miRNA) targets and corresponding mRNA targets. Real hsa_circ_0004781, which is identical to the native form of hsa_circ_0004781 without any exogenous sequences, was prepared through in vitro transcription by using a ribozyme and ion-pair reversed-phase high-performance liquid chromatography (IP-RP HPLC). The biological functions of hsa_circ_0004781 were evaluated using loss-of-function and gain-of-function approaches with circRNA expression plasmids and real hsa_circ_0004781.

**Results:**

Knockdown of hsa_circ_0004781 inhibited the proliferation and migration of PDAC cells, whereas its overexpression produced opposite effects. Hsa_circ_0004781 was identified as a sponge for miR-9-5p and miR-338-3p, and its expression was negatively correlated with that of these miRNAs. Among the targets of miR-9-5p and miR-338-3p, Kruppel-like factor 5 (*KLF5*) and a disintegrin and metalloproteinase domain 17 (*ADAM17*) were negatively correlated with survival in patients with PDAC and were inversely regulated by these miRNAs. Furthermore, real hsa_circ_0004781 exhibited the same effects as those of the circRNA expression plasmids.

**Conclusions:**

This study is the first to use real circRNAs to validate results obtained using circRNA expression plasmids. The results suggest that hsa_circ_0004781 functions as an oncogene, promoting the proliferation of PDAC cells through the miR-9-5p/*KLF5* and miR-338-3p/*ADAM17* axes. Therefore, hsa_circ_0004781 might be a therapeutic target for PDAC.

**Supplementary Information:**

The online version contains supplementary material available at 10.1186/s12935-025-03687-0.

## Introduction

Circular RNAs (circRNAs) are generated from precursor mRNA (pre-mRNA) through back-splicing, a process that forms a back-splicing junction (BSJ) and results in a closed-loop structure [1, 2]. CircRNAs have various biological functions [3], including acting as sponges for microRNAs (miRNAs) and competing with endogenous RNAs to bind miRNAs. In doing so, they regulate the expression of miRNA target mRNAs. In addition, circRNAs bind to proteins to inhibit their activity, serve as scaffolds for protein–protein interactions, and direct proteins to specific locations. CircRNAs also regulate transcription and alternative splicing and can undergo rolling circle translation. A growing number of studies has reported that circRNAs are associated with various diseases, including Alzheimer’s disease, neurological disorders, osteoarthritis, diabetes, cardiovascular diseases, and cancers. CircRNAs have been indicated to play crucial roles in tumor initiation, progression, and drug resistance [4, 5]. Unlike linear mRNAs, circRNAs possess a covalently closed circular structure that confers resistance to RNase R degradation, which enhances their stability [6].

Pancreatic ductal adenocarcinoma (PDAC) accounts for 90% of pancreatic cancers and is among the most aggressive solid malignancies. Because of challenges related to early diagnosis, high genetic heterogeneity, and drug resistance, the 5-year survival rate for PDAC is only approximately 5–7%. Therefore, additional research must be conducted to more comprehensively understand PDAC tumorigenesis and explore potential treatment strategies. Studies have reported that circRNAs can promote the proliferation, metastasis, and progression of PDAC through various mechanisms. For example, hsa_circ_0007590 binds to polypyrimidine tract-binding protein 1 (PTBP1), enhancing the expression of the m^6^A reader protein YTH N^6^-methyladenosine RNA-binding protein 2 (YTHDF2). This interaction results in the degradation of phosphatase and tensin homolog (*PTEN*) mRNA, thereby activating the phosphatidylinositol 3-kinase/AKT signaling pathway [7]. Additionally, hsa_circ_0000919 acts as a sponge for miR-186 and miR-326, preventing the degradation of high-mobility AT-hook 2 (*HMGA2*) mRNA by these miRNAs. This inhibition promotes PDAC cell proliferation, metastasis, and resistance to apoptosis [8]. The common Kirsten rat sarcoma (*KRAS*) mutation in PDAC cells is associated with circRNA expression. A *KRAS* mutation increases the expression of hsa_circ_0060665, which acts as a sponge for miR-1205, preventing the miR-1205-mediated degradation of Janus kinase 2 (*JAK2*). This process activates the JAK2/signal transduction and activator of transcription 3 (STAT3) signaling pathway, facilitating PDAC metastasis [9]. In addition, Zhao et al. reported that hsa_circ_0011385 promotes PDAC progression by forming a complex with mothers against decapentaplegic homolog 3 (SMAD3) and adaptor-related protein complex 2 subunit alpha 1 (AP2A1), facilitating the entry of mothers against decapentaplegic homolog 3 into endosomes and activating the transforming growth factor (TGF)-β signaling pathway [10].

In this study, we identified hsa_circ_0004781, a circRNA derived from exons 2–5 of the Pecanex 1 (*PCNX1*) gene, which is highly expressed in PDAC. Our results demonstrated that hsa_circ_0004781 might play a key role in regulating the proliferation of PDAC cells through the miR-9-5p/ Kruppel-like factor 5 (*KLF5*) and miR-338-3p/ disintegrin and metalloproteinase domain 17 (*ADAM17*) axes.

## Materials and methods

### Materials

The following materials were used in this study: a rabbit polyclonal anti-cyclin D1 (M-20) antibody from Santa Cruz Biotechnology (Santa Cruz, CA, USA); rabbit monoclonal anti-E-cadherin (24E10) and rabbit monoclonal anti-vimentin (D21H3) antibodies from Cell Signaling Technology (Danvers, MA, USA); and rabbit polyclonal anti-KLF5 (GTX103289), rabbit polyclonal anti-ADAM17 (GTX101358), rabbit polyclonal anti-GAPDH (GTX100118) antibodies, and anti-mouse immunoglobulin G horseradish peroxidase (GTX213111-01) and anti-rabbit immunoglobulin G horseradish peroxidase secondary antibodies (GTX213110-01) from GeneTex International (Hsinchu City, Taiwan).

### Cell culture

AsPC-1 and BxPC-3 human pancreatic adenocarcinoma cell lines were kindly provided by Prof. Shiow-Lin Pan from the Graduate Institute of Cancer Biology and Drug Discovery, Taipei Medical University. Both cell lines were cultured in Roswell Park Memorial Institute medium (Gibco, ThermoFisher Scientific, Waltham, MA, USA) supplemented with 10% fetal bovine serum and1% penicillin–streptomycin. The cells were cultured in a humidified incubator at 37 °C with 5% CO_2_.

### Plasmid construction

To construct the circRNA expression plasmid, pcDNA-ZKSCAN-c4781, complementary DNA (cDNA) encoding exons 2–5 of *PCNX1*, and part of intron 2 containing splicing acceptor sequences and part of intron 6 containing splicing donor sequences were synthesized and subcloned into the pcDNA3.1(+) ZKSCAN1 MCS Exon vector (Addgene plasmid no. 69901) by Genomics (New Taipei City, Taiwan). To generate the miRNA reporter plasmid, pmirGLO-c4781, cDNA encoding the miR-9-5p or miR-338-3p target sequence from hsa_circ_0004781, was synthesized and subcloned downstream of the firefly luciferase gene in the Dual-Luciferase miRNA Target Expression Vector (Promega, Madison, WI, USA) by MDBio (New Taipei City, Taiwan). The predicted miR-9-5p and miR-338-3p target sequences of hsa_circ_0004781 were 5′-GUUCACGGAUCAGCGAACCAAAGC-3′ and 5′-CUACACAGAGCACUUGAUGCUGGA-3′, respectively (Fig. 4A).

### Transient transfection

A fixed number of cells (5–10 × 10^5^ cells) were seeded in 3.5-cm culture dishes and transfected with siRNAs, miRNAs, or plasmids by using Lipofectamine RNAiMAX or Lipofectamine 3000 in accordance with the manufacturer’s instructions (Gibco, ThermoFisher Scientific) [11].

### Luciferase reporter assay

Cells were cotransfected with the pmirGLO-c4781 reporter plasmid and either a control miRNA mimic, miR-9-5p mimic, or miR-338-3p mimic (Supplementary Table S2). Total protein lysates were collected, and luciferase activity was measured using the Dual-Glo Luciferase Assay Kit (Promega) in accordance with the manufacturer’s instructions. Firefly luciferase and *Renilla* luciferase (internal control) activities were quantified using a SpectraMax iD3 Multi-Mode Microplate Reader (Molecular Devices, San Jose, CA, USA) [12].

### Cell viability assay

Cells (2–4 × 10⁴) were seeded in 96-well plates. After treatment, either 5 mg/mL of 3-(4,5-dimethylthiazol-2-yl)-2,5-diphenyltetrazolium bromide (MTT) or one-tenth volume of Cell Counting Kit-8 (CCK8) solution was added to the cells. These cells were then incubated for 2–4 h. For the MTT assay, the medium was removed, and 100 µL of dimethyl sulfoxide (DMSO) was added to dissolve MTT formazan crystals. Optical density was measured at 570 or 450 nm by using a SpectraMax iD3 Multi-Mode Microplate Reader (Molecular Devices, San Jose, CA).

### Wound healing assay

Cells (2–5 × 10^5^ per well) were seeded in 24-well plates. After treatment, a wound was created using a 200-µL pipette tip, and positions were marked on the culture plate with an ink marker. The cells were washed with phosphate-buffered saline, and fresh medium was added. Initial images (at 0 h) were captured under a microscope to record the baseline wound area. After 24 h, images were again captured to enable observation of cell migration and wound closure. The wound area was analyzed using the ImageJ Plugin developed by Alejandra Suarez-Arnedo (ImageJ/Fiji) from the provided link (https://github.com/AlejandraArnedo/Wound-healing-size-tool/wiki). Wound closure was quantified using the following formula: % wound closure = [(wound area at 0 h) − (wound area at 24 h)]/(wound area at 0 h) × 100% [13].

### Western blot analysis

Cells (1–2 × 10^6^) were seeded in 6-cm culture dishes. After treatment, total cellular protein lysates were collected using Gold Lysis Buffer (137 mM NaCl, 20 mM Tris at pH 7.9, 10 mM NaF, 1% Triton X-100, 10% glycerol, 5 mM EDTA, 1 mM EGTA, 1 mM phenylmethylsulfonyl fluoride, 10 µg/mL aprotinin, 10 µg/mL leupeptin, 1 mM sodium orthovanadate, 1 mM sodium pyrophosphate, and 100 µM β-glycerophosphate). Protein lysates (10–30 µg) were mixed with 5× loading buffer (335 mM Tris at pH 6.8, 10% sodium dodecyl sulfate, 0.15% bromophenol blue, 4.5% glycerol, and 1% β-mercaptoethanol) and heated to 95 °C for 5 min. The samples were then subjected to sodium dodecyl sulfate–polyacrylamide gel electrophoresis (PAGE). The proteins were transferred from the gel onto polyvinylidene difluoride membranes and incubated with a primary antibody and a secondary antibody conjugated to horseradish peroxidase. Protein bands were visualized using enhanced chemiluminescence kits (Amersham, Arlington, IL, USA) and imaged using the ImageQuant LAS4000 Imager system (GE Healthcare Life-Sciences, Taiwan Branch, Taipei, Taiwan) [14].

### Reverse transcription–polymerase chain reaction

Total RNA was extracted using Trizol RNA reagent (Invitrogen, ThermoFisher Scientific). Two micrograms of total RNA were used for reverse transcription (RT) with random primers and M-MLV Reverse Transcriptase (Applied Biosystems; ThermoFisher Scientific) to synthesize cDNA. Gene expression was amplified from the cDNA by using specific primers (Supplementary Table S1) and DreamTaq DNA polymerase in accordance with the manufacturer’s instructions (Fermentas; ThermoFisher Scientific). Polymerase chain reaction (PCR) was conducted in a Veriti 96-Well Thermal Cycler (Applied Biosystems, ThermoFisher Scientific) under the following conditions: 40 cycles of 95 °C for 30 s, 60 °C for 30 s, and 72 °C for 15 s [15].

### Quantitative PCR

Quantitative PCR (qPCR) was performed using SYBR Green PCR Master Mix (Applied Biosystems) in accordance with the manufacturer’s instructions. Each 20-µL reaction mixture contained 20 ng of cDNA, 0.2 µM of each primer, and 2× SYBR Green PCR Master Mix. Reactions were conducted using the StepOne Real-Time PCR System (Applied Biosystems). Primers employed in the qPCR were validated using BLAST to ensure specificity to their respective oligonucleotides and designed to span splice junctions for accurate mRNA measurements. The primer sequences are listed in Supplementary Table S1. The qPCR conditions were as follows: initial denaturation at 95 °C for 20 s, followed by 40 cycles of 95 °C for 3 s and 60 °C for 30 s. Melting curve analysis was performed in three steps: 95 °C for 15 s, 60 °C for 1 min, and 95 °C for 15 s. Relative mRNA expression levels were determined using the Δ^Ct^ method, and GAPDH mRNA was used as the internal control for normalization [16].

### miRNA detection

For miRNA detection, 2 µg of total RNA was used to synthesize polyA-tailed miRNA, which was subsequently reverse-transcribed into cDNA by using an Anchor oligo dT adaptor sequence with the qSTAR miRNA cDNA synthesis kit (Level Bio, New Taipei City, Taiwan). A mixture of 2 µL of cDNA and 2× SYBR Green PCR Master Mix was prepared. PCR was conducted on the StepOne Real-Time PCR System (Applied Biosystems). Relative miRNA expression levels were determined using the Ct method (Δ^Ct^ formula), and U6 was used as the positive control for normalization.

### *Tetrahymena* group I intron ribozyme design, in vitro transcription, and denaturing urea–PAGE

Real hsa_circ_0004781 was generated in vitro by using the *Tetrahymena thermophila* group I intron ribozyme with some modifications [17]. Briefly, cDNA containing the T7 promoter, ribozyme, and hsa_circ_0004781 was synthesized and inserted into the *Kpn I*/*BamH I* sites of the pUC57-Kan vector by GenScript Biotech Corporation (Piscataway, NJ, USA). For in vitro transcription (IVT), a DNA template encoding the T7 promoter, ribozyme, and hsa_circ_0004781 was amplified using the aforementioned vector through PCR and purified using the NucleoSpin Gel and PCR Clean-up Kit (MACHEREY-NAGEL GmbH & Co., Düren, Germany). The DNA template was then used to synthesize pre-mRNA transcripts in vitro with the HiScribe T7 High Yield RNA Synthesis Kit (New England Biolabs, Ipswich, MA, USA), and an additional guanosine monophosphate (GMP) was used as cofactor. After DNase I digestion of the DNA template, IVT products were analyzed using 5% PAGE with 7 M urea at 55 °C. The gels were poststained with GelRed dye (Merck KGaA, Darmstadt, Germany) to visualize RNA bands.

### Ion-pair reversed-phase high-performance liquid chromatography (IP-RP HPLC)

IVT products were separated through IP-RP HPLC [18, 19] by using the Agilent 1290 Infinity II LC System (Agilent Technologies, Santa Clara, CA, USA) with PLRP-S HPLC columns (length, 250 mm; i.d., 10 mm; particle size, 5 μm; pore size, 4000 Å). A linear gradient was applied using mobile phase A (100 mM hexylammonium acetate in 40% acetonitrile) and mobile phase B (100 mM hexylammonium acetate in 50% acetonitrile) at a flow rate of 2.0 mL/min for 40 min. The column oven was maintained at 60 °C, and RNA was detected at 260 nm. The high-performance liquid chromatography (HPLC) peak corresponding to hsa_circ_0004781 was collected and precipitated by adjusting the NaCl concentration to 0.25 M, followed by the addition of 2.5 volumes of ethanol and incubation at − 80 °C for at least 2 h.

### Statistical analysis

Data are presented as the mean ± standard error (SE) from independent experiments, as indicated. Statistical significance was determined using a one-way Student’s *t*-test. Significance is denoted by an asterisk (*) or hashtag (^#^), and a *p* value of < 0.05 was considered significant. Data analyses were conducted using GraphPad Prism 9 software (La Jolla, CA, USA).

## Results

### Identification of hsa_circ_0004781 in PDAC cells

**Fig. 1:**
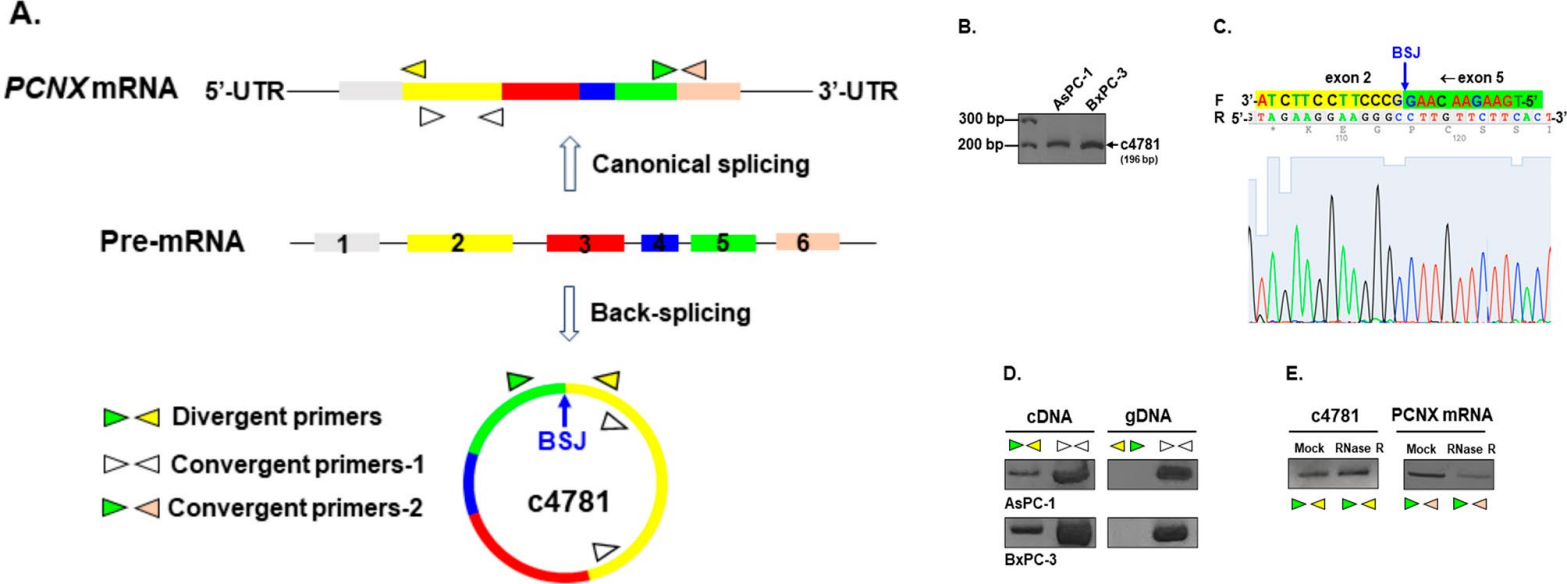
Identification of hsa_circ_0004781 in PDAC cells. (**A**) Schematic illustrating the canonical and back-splicing of *PCNX1* pre-mRNA, presenting three paired primer annealing sites for *PCNX1* mRNA and hsa_circ_0004781. (**B**) Total RNA from ASPC-1 and BXPC-3 cells was extracted, and hsa_circ_0004781 (c4781) was amplified through RT-PCR by using divergent primers, followed by agarose gel electrophoresis. (**C**) The hsa_circ_0004781 band was extracted from the gel, and the sequence of the BSJ region was determined through Sanger sequencing. F, forward strand; R, reverse strand. (**D**) cDNA and gDNA prepared from AsPC-1 and BxPC-3 cells were used to amplify the BSJ region of hsa_circ_0004781 and exon 2 of the *PCNX1* gene by using divergent primers and convergent primer 1, respectively. (**E**) Total RNA extracted from AsPC-1 cells treated with or without RNase R was used to amplify the BSJ region of hsa_circ_0004781 and exon 2 of *PCNX1* mRNA through RT-PCR with divergent primers and convergent primer 2, respectively

We initially screened for circRNAs that were highly expressed in PDAC cells by using the GSE79634 and GSE69362 databases. Our analysis revealed that the expression of hsa_circ_0004781 was significantly higher in PDAC tissues than in normal pancreatic tissues, with a log2 fold change of 1.906 and 0.662 in the GSE79634 and GSE69362 databases, respectively (Supplementary Fig. S1). Hsa_circ_0004781, which was predicted to be 451 nucleotides long, is formed by the back-splicing of exons 2–5 of the *PCNX1* gene. To detect hsa_circ_0004781 in PDAC cells, we performed RT-PCR on AsPC-1 and BxPC-3 cell lines by using divergent primers (Fig. 1A). Both the AsPC-1 and the BxPC-3 cells expressed hsa_circ_0004781 (Fig. 1B). To confirm that hsa_circ_0004781 originated from back-splicing, we extracted RT-PCR products from gels and sequenced the BSJ region. Sanger sequencing results confirmed the presence of the BSJ (Fig. 1C). In addition, we designed convergent primer set 1 to amplify exon 2 of *PCNX1* and hsa_circ_0004781 and primer set 2 to amplify the exon 5–exon 6 junction of *PCNX1* mRNA (Fig. 1A). The divergent primers amplified template cDNA but not genomic DNA, indicating that hsa_circ_0004781 originated from RNA splicing instead of genomic DNA (Fig. 1D). Furthermore, in contrast to *PCNX1* mRNA, hsa_circ_0004781 was resistant to RNase R degradation, which confirms that it has a circular structure (Fig. 1E).

### Promotion of proliferation and migration of PDAC cells by hsa_circ_0004781

**Fig. 2:**
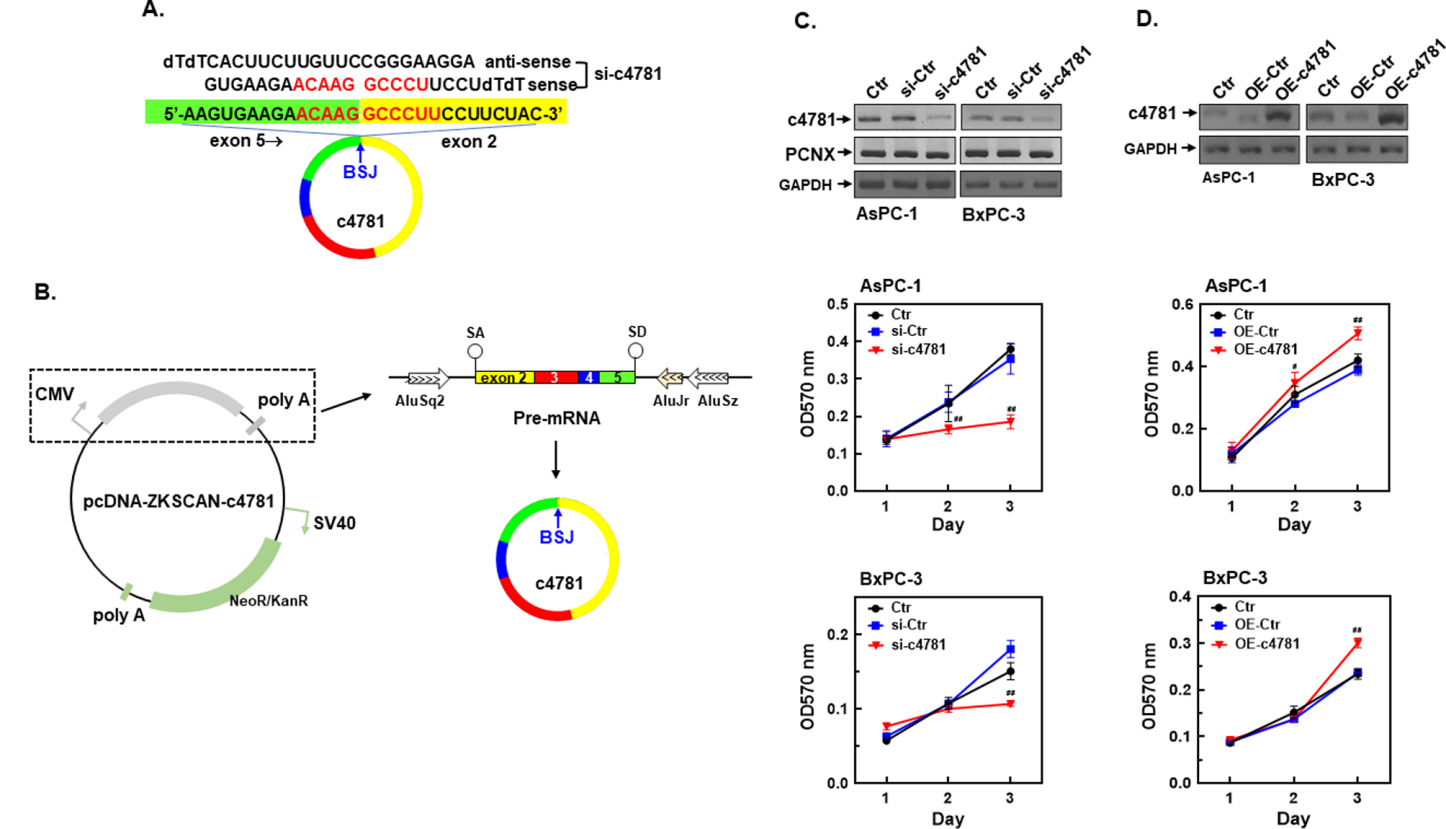
Effects of the knockdown and overexpression of hsa_circ_0004781 on PDAC cell proliferation. (**A**) hsa_circ_0004781 siRNA (si-c4781) was designed to target the BSJ region of hsa_circ_0004781 (c4781), and si-c4781 sequences are presented. (**B**) The pcDNA3.1(+) ZKSCAN1 MCS Exon vector was used to construct the pcDNA-ZKSCAN-c4781 plasmid, which transcribed a pre-mRNA containing exons 2–5 of *PCNX1* mRNA, the partial splicing acceptor (SA) and splicing donor (SD) intronic regions of the *PCNX1* gene, and Alu sequences of the *ZKSCAN1* gene. (**C**, **D**) AsPC-1 and BxPC-3 cells were transfected with (**C**) control siRNA (si-Ctr) or si-c4781 and (**D**) the control vector (OE-Ctr) or pcDNA-ZKSCAN-c4781 plasmid (OE-c4781) for 1–3 days. Total RNA was extracted, and the expression of hsa_circ_0004781 was determined through RT-PCR. Cell proliferation was examined using an MTT assay. Values are presented as mean ± SE, *n* > 3. ^##^*p* < 0.0001 compared with si-Ctr or OE-Ctr

To investigate the role of hsa_circ_0004781 in PDAC cells, we designed a specific siRNA, si-c4781, that was complementary to the BSJ region of hsa_circ_0004781 to knockdown hsa_circ_0004781 (Fig. 2A). We confirmed its binding specificity by using BLAST. Transfection of AsPC-1 and BxPC-3 cells with si-c4781 significantly reduced the expression of hsa_circ_0004781 and markedly inhibited the proliferation of both cell lines (Fig. 2C). To overexpress hsa_circ_0004781, we cloned exons 2–5 of *PCNX1* cDNA into the pcDNA3.1(+) ZKSCAN1 MCS Exon vector, named pcDNA-ZKSCAN-c4781. This vector expressed linear pre-mRNA flanked by Alu sequences upstream and downstream, which allowed stem-loop formation to facilitate back-splicing and generate hsa_circ_0004781 (Fig. 2B). In addition, transfection of pcDNA-ZKSCAN-c4781 into AsPC-1 and BxPC-3 cells significantly increased hsa_circ_0004781 expression and cell proliferation (Fig. 2D).

**Fig. 3:**
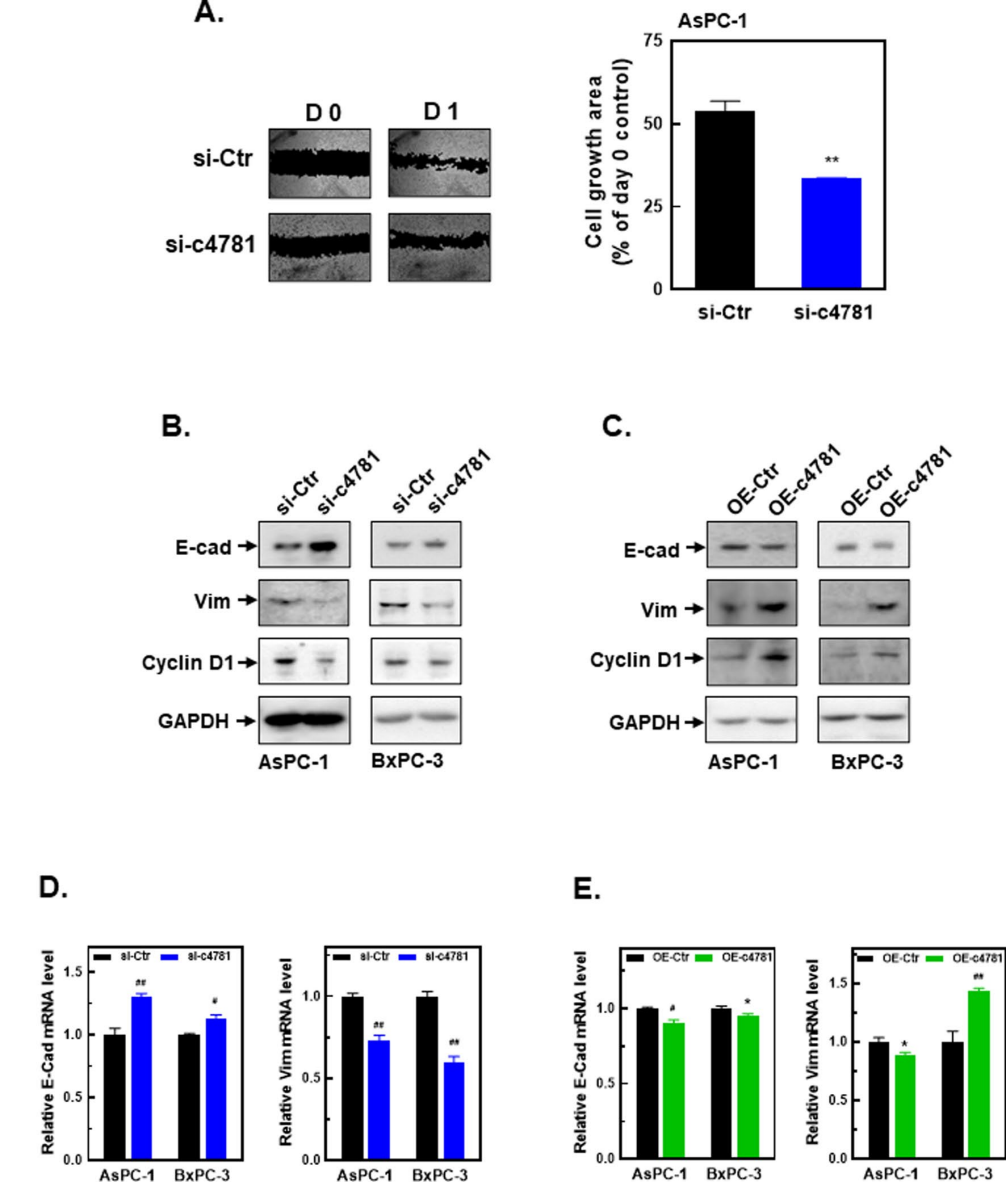
Effects of the knockdown and overexpression of hsa_circ_0004781 on the cell migration and expression of EMT-related marker genes in PDAC cells. (**A**) AsPC-1 cells were transfected with control siRNA (si-Ctr) or hsa_circ_0004781 siRNA (si-c4781) for 24 h, and the number of migrating cells was determined by performing a wound healing assay. A representative photo and quantified data are presented. Each value represents the mean ± SE of three independent experiments. **p < 0.01 vs. si-Ctr. (**B-E**)AsPC-1 and BxPC-3 cells were transfected with (**B**, **D**) control siRNA (si-Ctr) or si-c4781 and (**C**, **E**) the control vector (OE-Ctr) or the pcDNA-ZKSCAN-c4781 plasmid (OE-c4781) for 24 h. (**B**, **C**) Total proteins were extracted, and protein expression was determined through a Western blot assay. (**D**, **E**) Total RNA was extracted, and mRNA expression was determined through RT-qPCR. Values are presented as the mean ± SE from three independent experiments. *p < 0.05, ^#^*p* < 0.001, ^##^*p* < 0.0001 vs. si-Ctr or OE-Ctr. E-cad, E-cadherin; Vim, vimentin

We subsequently examined whether hsa_circ_0004781 affected the migration of PDAC cells and expression of epithelial–mesenchymal transition (EMT)-related genes. In a wound healing assay, the knockdown of hsa_circ_0004781 by si-c4781 significantly inhibited the migration of AsPC-1 cells (Fig. 3A). Furthermore, the knockdown of hsa_circ_0004781 increased the protein and mRNA expression of E-cadherin but suppressed the mRNA and protein expression of vimentin and cyclin D1 (Fig. 3B, D). By contrast, the overexpression of hsa_circ_0004781 by pcDNA-ZKSCAN-c4781 reduced the protein and mRNA expression of E-cadherin but increased the mRNA and protein expression of vimentin and cyclin D1 in both AsPC-1 and BxPC-3 cells (Fig. 3C, E). These results demonstrated that hsa_circ_0004781 promoted cell proliferation and migration by regulating the expression of cyclin D1 and EMT-related genes, suggesting that hsa_circ_0004781 acts an oncogene in PDAC cells.

### Hsa_circ_0004781 as a sponge for miR-9-5p and miR-338-3p

**Fig. 4:**
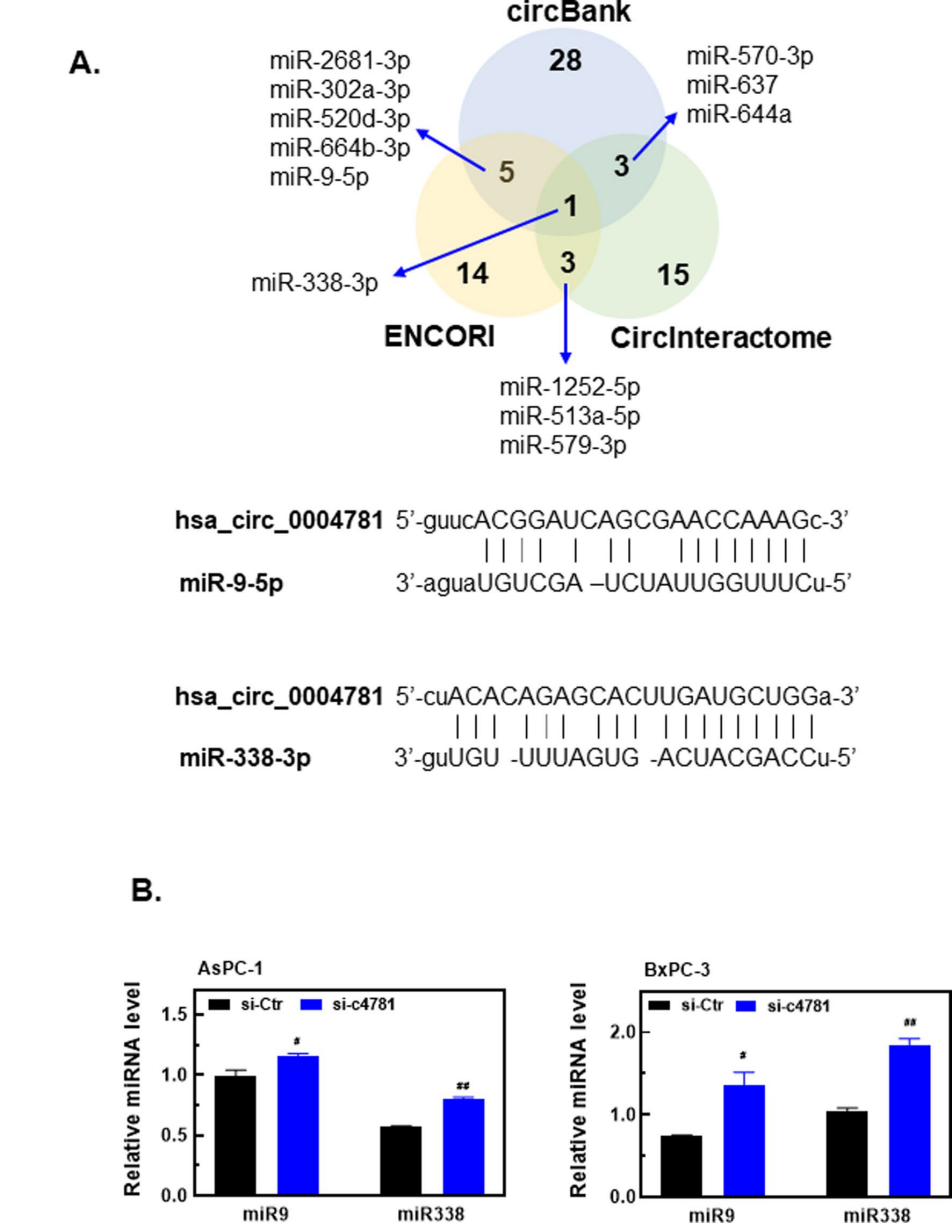
Effects of the knockdown of hsa_circ_0004781 on the expression of miR-9-5p and miR-338-3p in PDAC cells. (**A**) Potential target miRNAs of hsa_circ_0004781 were predicted using ENCORI, circBank, and CircInteractome. miR-9-5p and miR-338-3p were selected on the basis of their downregulation in PDAC. Bioinformatics analysis predicted potential miR-9-5p and miR-338-3p binding sites on hsa_circ_0004781. (**B**) AsPC-1 and BxPC-3 cells were transfected with control siRNA (si-Ctr) or hsa_circ_0004781 siRNA (si-c4781) for 24 h. Total RNA was extracted, and the expression of miR-9-5p and miR-338-3p were determined through RT-qPCR. Values are presented as the mean ± SE from three independent experiments. ^#^*p* < 0.001, ^##^*p* < 0.0001 vs. si-Ctr

Because argonaute-2, a catalytic component of the RNA-induced silencing complex, was predicted to bind hsa_circ_0004781 by Circinteractome (https://circinteractome.nia.nih.gov/), we investigated whether hsa_circ_0004781 functions as a sponge for miRNAs. Target miRNAs of hsa_circ_0004781 were predicted using ENCORI (https://rnasysu.com/encori/), CircInteractome, and circBank (http://www.circbank.cn/). miRNAs identified by at least 2 of these 3 platforms were shortlisted, with 12 candidates identified (Fig. 4A, upper). Among these, miR-9-5p and miR-338-3p were aberrantly downregulated in PDAC [20–22] and predicted to be potential targets of hsa_circ_0004781. Bioinformatics analysis revealed that hsa_circ_0004781 had putative complementary binding sites for miR-9-5p and miR-338-3p (Fig. 4A, bottom). Knockdown of hsa_circ_0004781 significantly increased the expression of miR-9-5p and miR-338-3p in both AsPC-1 and BxPC-3 cells (Fig. 4B), suggesting that hsa_circ_0004781 acts as a sponge for miR-9-5p and miR-338-3p in PDAC.

**Fig. 5:**
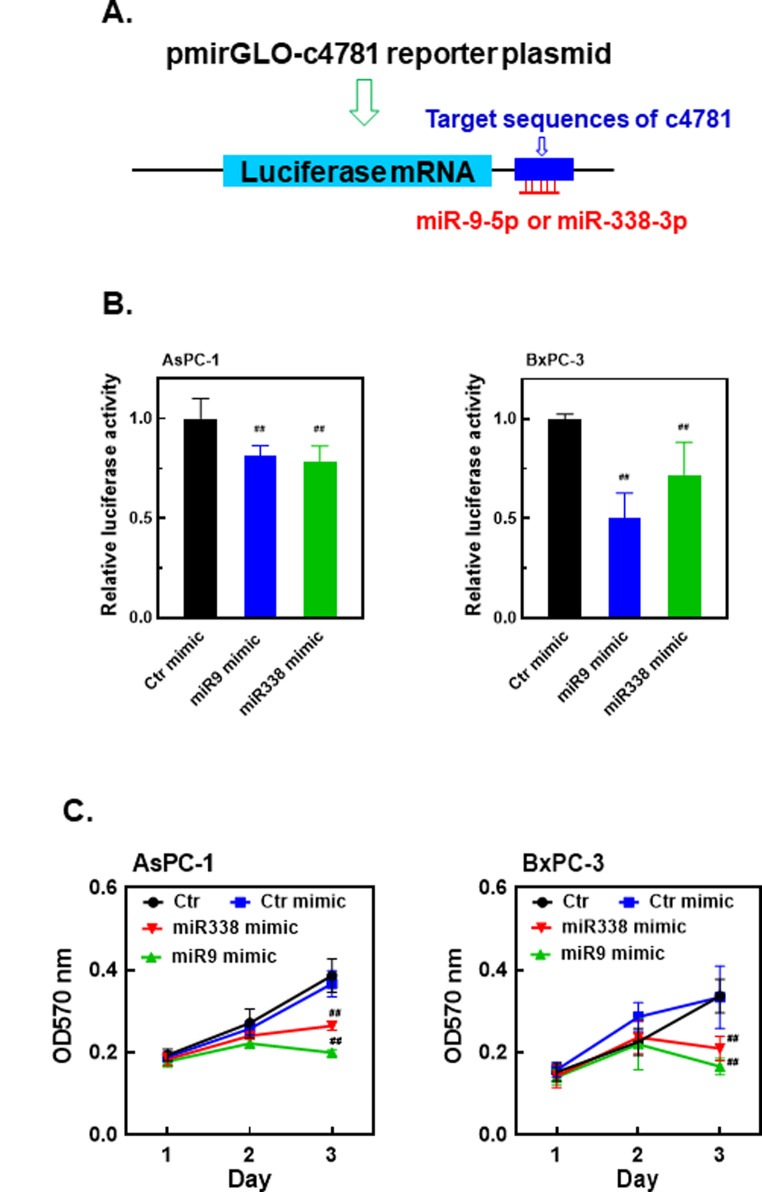
Effects of miR-9-5p and miR-338-3p mimics on luciferase mRNA degradation and PDAC cell proliferation. (**A**) The 3′ UTR of luciferase mRNA contained predicted miR-9-5p and miR-338-3p target sequences from hsa_circ_0004781 (c4781). (**B**) AsPC-1 and BxPC-3 cells were cotransfected with the pmirGLO-c4781 reporter plasmid and either the control miRNA mimic (Ctr mimic), miR-9-5p mimic (miR9 mimic), or miR-338-3p mimic (miR338 mimic) for 24 h. Total cell lysates were collected. Luciferase activity was measured using a dual luciferase assay kit, and values were normalized to *Renilla* luciferase activity. Each value represents the mean ± SE of three independent experiments. ^##^*p* < 0.0001 vs. the Ctr mimic. (**C**) AsPC-1 and BxPC-3 cells were transfected with the control miRNA mimic (Ctr mimic), miR-9-5p mimic (miR9 mimic), or miR-338-3p mimic (miR338 mimic) for 1–3 days. Cell proliferation was determined by performing an MTT assay. Values are presented as mean ± SE. ^##^*p* < 0.0001, compared with the Ctr mimic

To verify whether miR-9-5p or miR-338-3p binds to hsa_circ_0004781 and induces the degradation of luciferase mRNA through the argonaute pathway, we constructed the pmirGLO-c4781 luciferase reporter vector. We inserted hsa_circ_0004781 sequences complementary to miR-9-5p or miR-338-3p into the 3′ untranslated region of the luciferase gene in the pmirGLO-c4781 reporter plasmid (Figs. 4A and 5A). AsPC-1 and BxPC-3 cells were cotransfected with the pmirGLO-c4781 reporter plasmid and either the miR-9-5p or miR-338-3p mimic. The miR-9-5p and miR-338-3p mimics both markedly reduced luciferase activity (Fig. 5B), indicating that miR-9-5p and miR-338-3p bind to hsa_circ_0004781 sequences, resulting in luciferase mRNA degradation. To examine whether miR-9-5p and miR-338-3p inhibit PDAC cell proliferation, we transfected cells with miR-9-5p and miR-338-3p mimics. Both the miR-9-5p and miR-338-3p mimics significantly inhibited the proliferation of AsPC-1 and BxPC-3 cells (Fig. 5C). These results suggest that hsa_circ_0004781 promotes cell proliferation by sponging miR-9-5p and miR-338-3p, thereby reducing the levels of free miR-9-5p and miR-338-3p in PDAC cells.

### Upregulation of hsa_circ_0004781 of the target gene expression of miR-9-5p and miR-338-3p

**Fig. 6:**
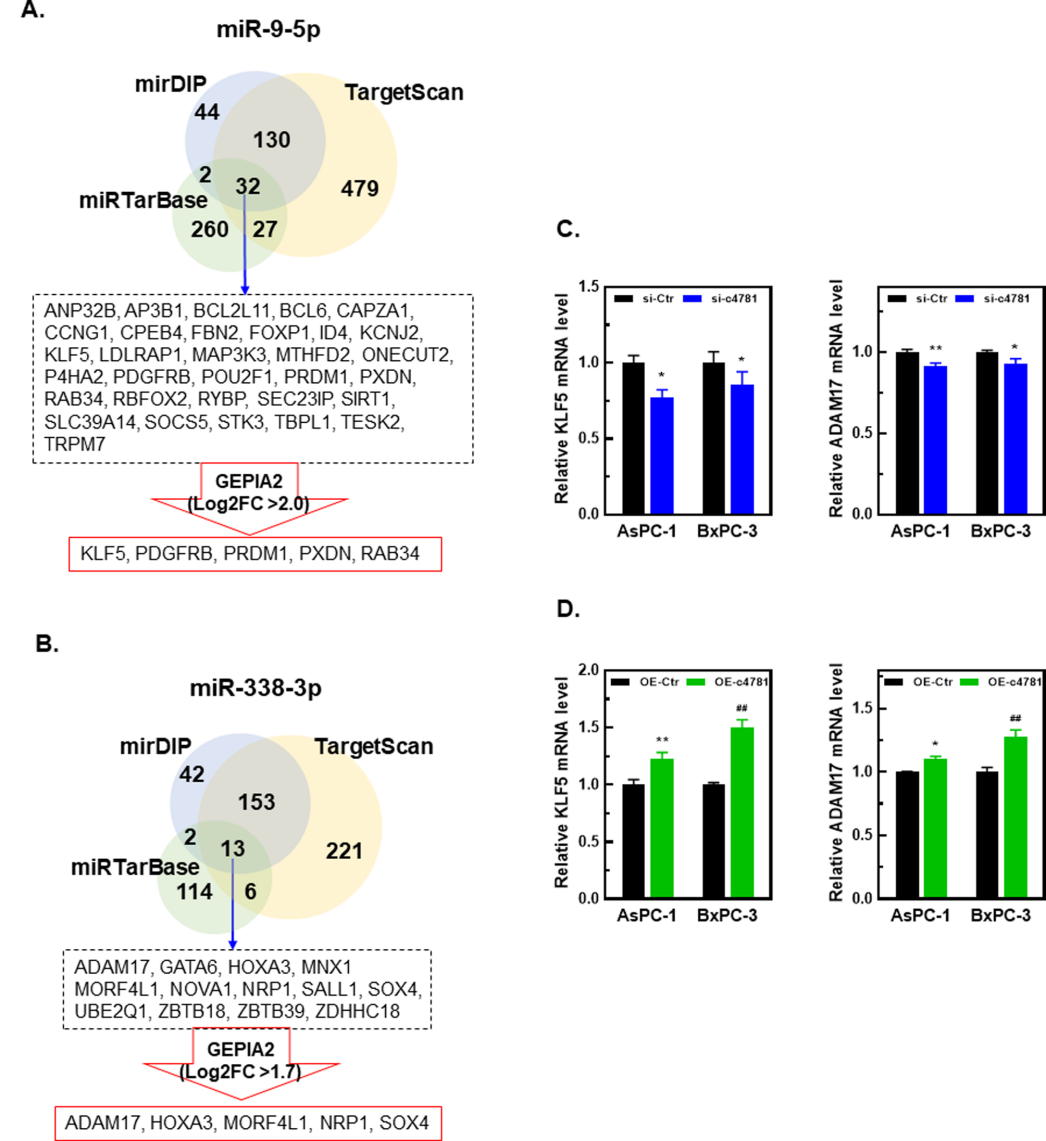
Effects of hsa_circ_0004781 on miR-9-5p and miR-338-3p target gene expression in PDAC cells. (**A**, **B**) Potential target mRNAs of (**A**) miR-9-5p and (**B**) miR-338-3p were predicted using mirDIP, miRTarBase, and TargetScan. Five upregulated targets were selected on the basis of the findings of GEPIA2 database analysis. (**C**) AsPC-1 and BxPC-3 cells were transfected with control siRNA (si-Ctr) or hsa_circ_0004781 siRNA (si-c4781) for 24 h. (**D**) AsPC-1 and BxPC-3 cells were transfected with a control vector (OE-Ctr) or pcDNA-ZKSCAN-c4781 plasmid (OE-c4781) for 24 h. (**C**, **D**) Total RNA was extracted, and the expression of miR-9-5p and miR-338-3p target mRNAs was determined through RT-qPCR. Values are presented as the mean ± SE from three independent experiments. *p < 0.05, **p < 0.01, ^##^*p* < 0.0001 vs. si-Ctr or OE-Ctr

We used three platforms, namely mirDIP (https://ophid.utoronto.ca/mirDIP/), miRTarBase (https://mirtarbase.cuhk.edu.cn/~miRTarBase/miRTarBase_2022/php/index.php), and TargetScan (https://www.targetscan.org/vert_80/), to predict potential target genes of miR-9-5p and miR-338-3p. From the 3 platforms, 32 candidate target genes were identified for miR-9-5p (Fig. 6A) and 13 for miR-338-3p (Fig. 6B). The five most highly expressed target genes for each miRNA, according to data from the GEPIA2 clinical database (http://gepia2.cancer-pku.cn/#index, PDAC vs. normal pancreas tissues), were selected for further analysis. The potential target genes of miR-9-5p were *KLF5*, *PDGFRB*, *PRDM1*, *PXDN*, and *RAB34*, and those of miR-338-3p were *ADAM17*, *HOXA3*, *MORF4L1*, *NRP1*, and *SOX4* (Fig. 6A and B; Supplementary Fig. S2).

To examine whether hsa_circ_0004781 regulates the expression of miR-9-5p and miR-338-3p target genes, we knocked down or overexpressed hsa_circ_0004781 and detected the expression of the 10 potential target genes through RT-qPCR. Knockdown of hsa_circ_0004781 (si-c4781) in AsPC-1 and BxPC-3 cells significantly reduced the expression of the miR-9-5p target genes *KLF5* (Fig. 6C, left panel) and *PXDN* (Supplementary Fig. S3A, left panel), the miR-338-3p target gene *ADAM17* (Fig. 6C, right panel), and the other four target genes (Supplementary Fig. S3A, right panels) in both cell lines. By contrast, the overexpression of hsa_circ_0004781 in AsPC-1 and BxPC-3 cells significantly increased the expression of *KLF5* and *ADAM17* (Fig. 6D) and that of the other four target genes of both miR-9-5p and miR-338-3p (Supplementary Fig. S3B).

**Fig. 7:**
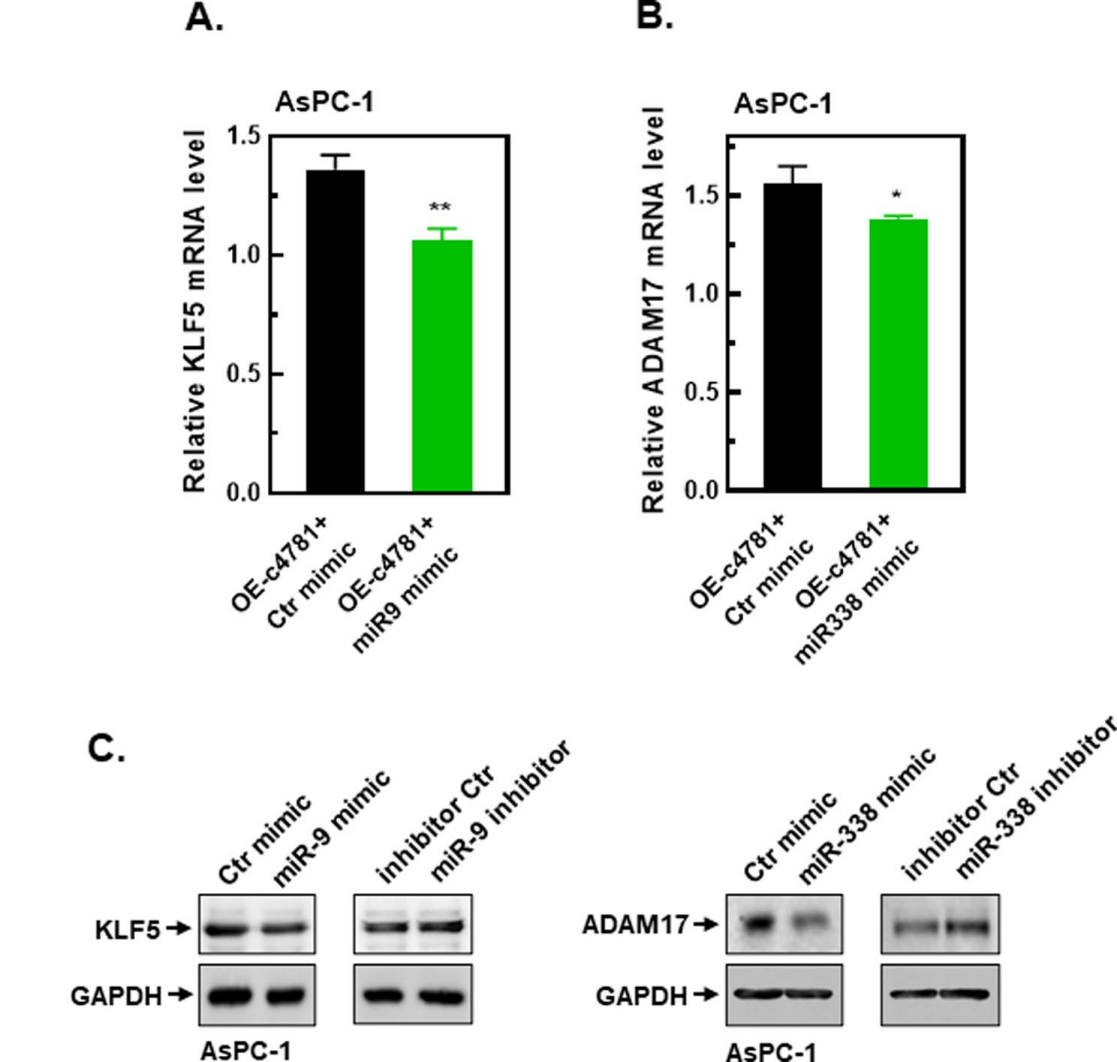
Effects of miR-9-5p and miR-338-3p on target gene expressions in PDAC cells. (**A**, **B**) AsPC-1 cells were cotransfected with the pcDNA-ZKSCAN-c4781 plasmid (OE-c4781) and either the control mimic (Ctr mimic), miR-9-5p mimic (miR9 mimic), or miR-338-3p mimic (miR338 mimic) for 24 h. Total RNA was extracted, and the expression of (**A**) miR-9-5p and (**B**) miR-338-3p target mRNAs was determined through RT-qPCR. Target gene expression levels were normalized to those of the control (no treatment). Values are presented as the mean ± SE from three independent experiments. *p < 0.05, **p < 0.01 vs. the Ctr mimic. (**C**) AsPC-1 cells were transfected with mimics or inhibitors of miR-9-5p and miR-338-3p for 24 h. Total cell protein lysates were collected, and the protein expression of KLF5 and ADAM17 was determined through Western blot analysis

To determine whether the upregulation of the 10 potential target genes induced by hsa_circ_0004781 could be reversed by miR-9-5p or miR-338-3p, we cotransfected AsPC-1 cells with the pcDNA-ZKSCAN-c4781 plasmid (OE-c4781) and either the miR-9-5p or miR-338-3p mimic. The miR-9-5p mimic significantly reduced the expression of *KLF5* (Fig. 7A) and of the other four target genes (Supplementary Fig. S4A), whereas the miR-338-3p mimic markedly inhibited the expression of *ADAM17* (Fig. 7B), *HOXA3*, and *NRP1* (Supplementary Fig. S4B) induced by hsa_circ_0004781. These findings suggest that *KLF5* and *PXDN* are significantly upregulated through the hsa_circ_0004781/miR-9-5p axis, and *ADAM17*, *HOXA3*, and *NRP1* are significantly upregulated through the hsa_circ_0004781/miR-338-3p axis.

### *KLF5* and *ADAM17* as the most crucial targets of miR-9-5p and miR-338-3p, respectively

We analyzed the correlation between the expression of target genes (*KLF5*, *PXDN*, *ADAM17*,* HOXA3*, and *NRP1*) and survival of patients with PDAC by using the GEPIA2 database. Among these targets, only *KLF5* and *ADAM17* were negatively correlated with patient survival (Supplementary Fig. S5), indicating that *KLF5* and *ADAM17* are the most crucial targets in the hsa_circ_0004781/miR-9-5p axis and hsa_circ_0004781/miR-338-3p axis, respectively. To confirm whether KLF5 and ADAM17 protein levels are regulated by miR-9-5p and miR-338-3p, we transfected AsPC-1 cells with mimics or inhibitors of miR-9-5p and miR-338-3p. The miR-9-5p and miR-338-3p mimics reduced the protein levels of KLF5 and ADAM17, respectively, whereas the miR-9-5p and miR-338-3p inhibitors increased the protein levels of KLF5 and ADAM17, respectively (Fig. 7C). These results suggest that the hsa_circ_0004781/miR-9-5p/*KLF5* and hsa_circ_0004781/miR-338-3p/*ADAM17* axes play crucial roles in the progression of PDAC.

### Generation of real hsa_circ_0004781 by ribozymes and purification of real hsa_circ_0004781 by IP-RP HPLC

**Fig. 8:**
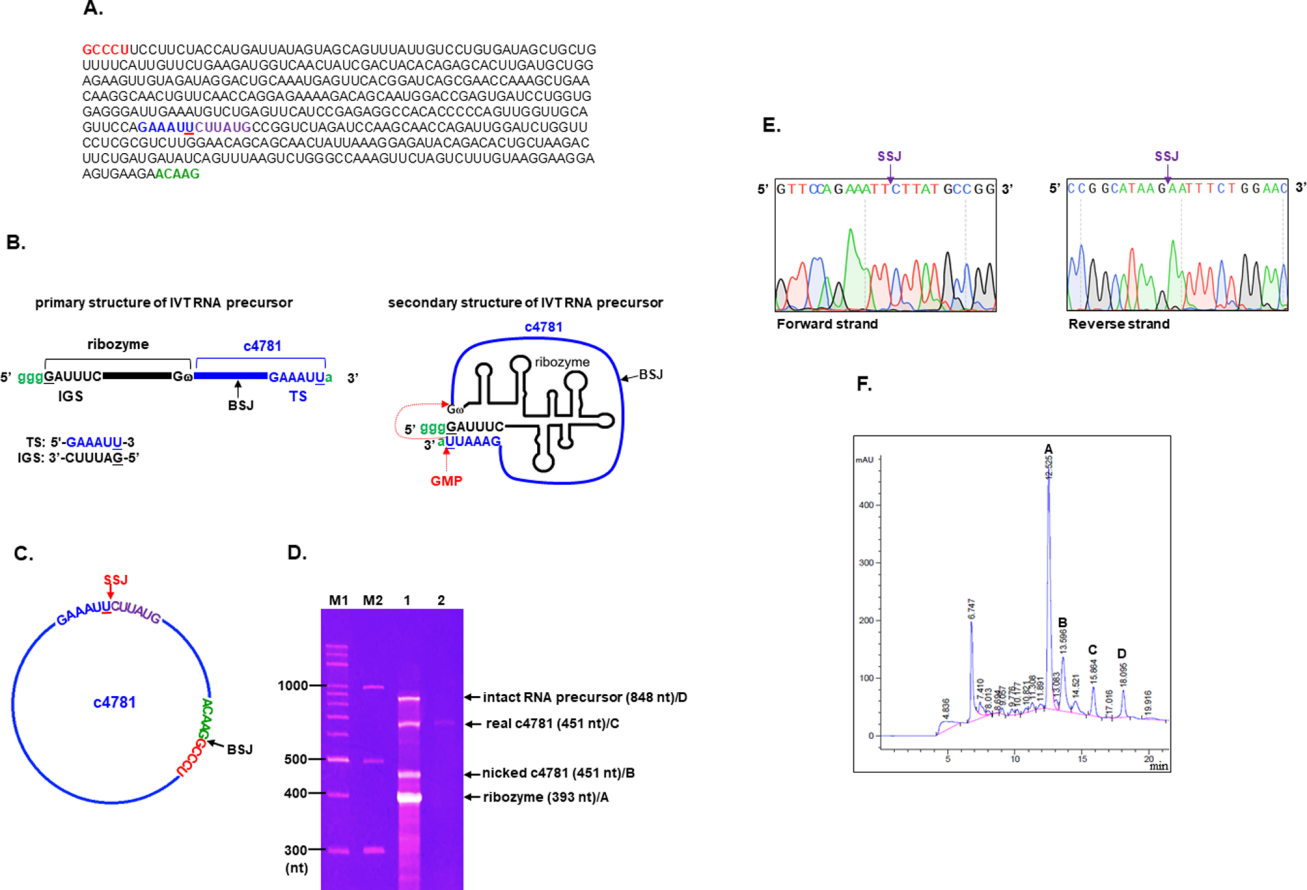
Generation and purification of real hsa_circ_0004781. (**A**) The sequences of hsa_circ_0004781 are presented, and the ribozyme target sequence is highlighted in blue. (**B**) Schematics for the primary and secondary structures of the IVT RNA precursor containing the *Tetrahymena* group I intron ribozyme and hsa_circ_0004781 (c4781). The 3′ target sequence (TS) is complementary to the 5′ internal guide sequence (IGS), forming a G·U wobble base pair. GMP induces self-splicing ligation through two transesterification reactions, forming real hsa_circ_0004781. (**C**) The self-splicing junction (SSJ) formed during ligation differs from the natural BSJ. (D, F) IVT products were (**D**) subjected to denaturing urea–PAGE and stained with GelRed dye or (**F**) separated through IP-RP HPLC. M1, DNA markers; M2, RNA markers; lane 1, IVT products; lane 2, peak C from HPLC. (**E**) Band C from urea–PAGE was extracted, and RNA was amplified at the SSJ region through RT-PCR. Sequences of forward and reverse strands were determined through Sanger sequencing

To confirm the findings obtained using circRNA expression plasmids, we generated real hsa_circ_0004781 in vitro through self-splicing ligation by using the *Tetrahymena* group I intron ribozyme. This in vitro-generated circRNA, referred to as real hsa_circ_0004781, is identical to the native form of hsa_circ_0004781 without any exogenous sequences. A DNA template containing a T7 promoter, the ribozyme, and hsa_circ_0004781 sequences was transcribed into RNA precursors through IVT. We designed ribozyme target sequences (5′-GAAAUU-3′) by using nucleotides 281–286 of hsa_circ_0004781 (Fig. 8A) and positioned them at the 3′ end of the RNA precursor. The target sequence was complementary to the internal guide sequence (5′-GAUUUC-3′) located at the 5′ end of the RNA precursor (Fig. 8B). A wobble base pair was formed between the underlined U in the target sequence and G in the internal guide sequence. During IVT, additional guanosine monophosphate initiated self-splicing ligation through two consecutive transesterification reactions, producing real hsa_circ_0004781 (Fig. 8C). The self-splicing junction (SSJ) differed from the natural BSJ.

To verify the generation of real hsa_circ_0004781 during IVT, we separated IVT products by using denaturing urea–PAGE. Four major bands corresponding to the intact RNA precursor (848 nt), nicked hsa_circ_0004781 (451 nt), real hsa_circ_0004781 (451 nt), and the ribozyme (393 nt), were detected (Fig. 8D). Because of its circular structure, real hsa_circ_0004781 was expected to migrate more slowly than the linear form of nicked hsa_circ_0004781 was on denaturing urea–PAGE. Therefore, we extracted the RNA band between 600 and 700 nucleotides, amplified the SSJ region through RT-PCR, and determined the sequences of both forward and reverse strands through Sanger sequencing. The sequence data for the SSJ region confirmed the generation of real hsa_circ_0004781 without any exogenous sequences (Fig. 8E). Next, we purified and collected real hsa_circ_0004781 through IP-RP HPLC. Each HPLC peak from A to D was collected, precipitated, and analyzed using denaturing urea–PAGE. Peak C was identified as real hsa_circ_0004781 (Fig. 8F), and we subsequently collected additional real hsa_circ_0004781 for further experiments.

### Real hsa_circ_0004781 downregulation of the expression of miR-9-5p and miR-338-3p and upregulation of the expression of *KLF5* and *ADAM17*

**Fig. 9:**
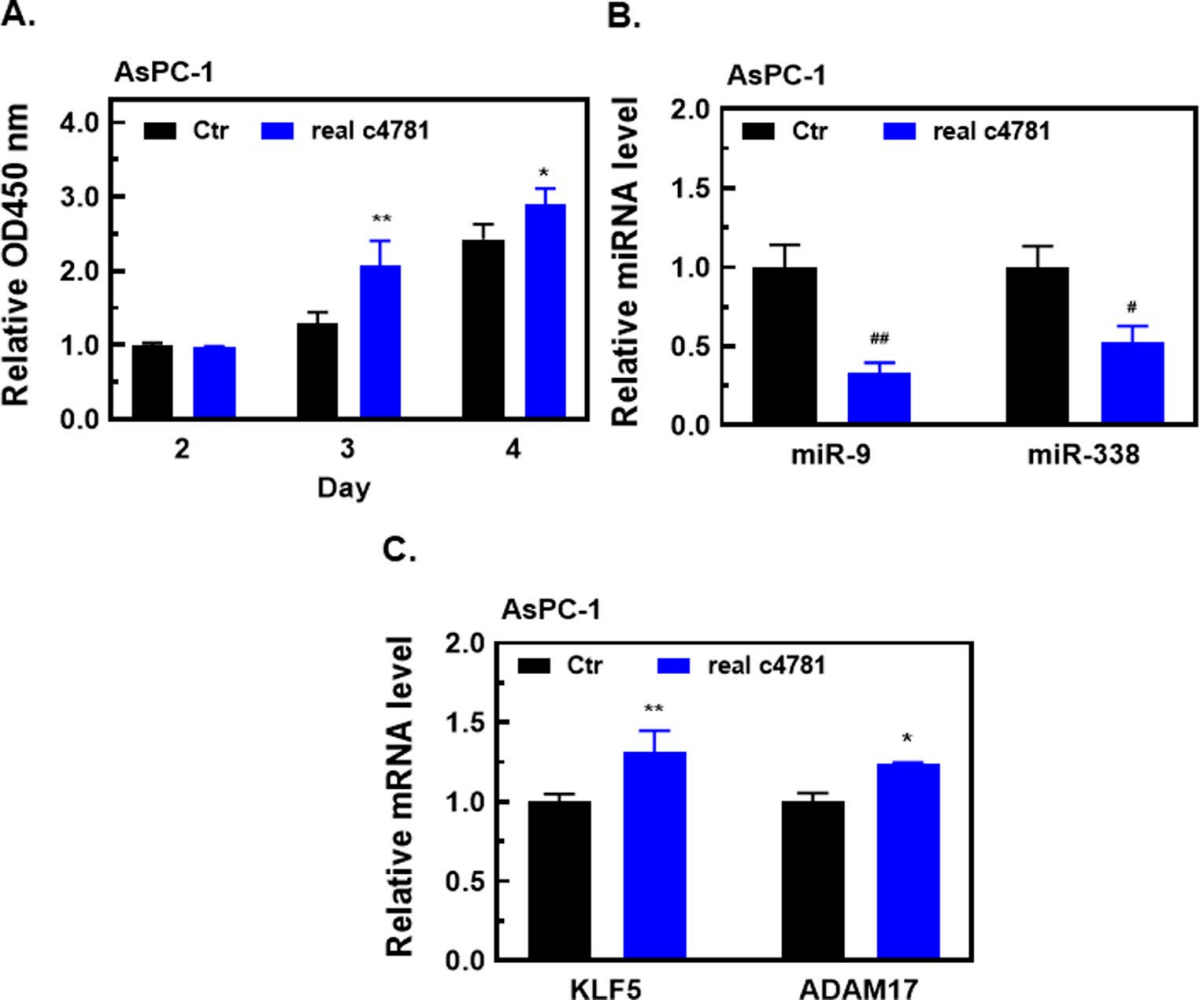
Effects of real hsa_circ_0004781 on cell proliferation and the expression of miR-9-5p, miR-338-3p, *KLF5*, and *ADAM17* in PDAC cells. AsPC-1 cells were transfected with control RNA (Ctr) or real hsa_circ_0004781 (real c4781) for 24 h. (**A**) Cell proliferation was determined using a CCK-8 assay. (**B**, **C**) Total RNA was extracted, and the expression of (**B**) miR-9-5p and miR-338-3p as well as (C) *KLF5* and *ADAM17* mRNA was detected through RT-qPCR. Values are presented as the mean ± SE from three independent experiments. *p < 0.05, **p < 0.01, ^#^*p* < 0.001, ^##^*p* < 0.0001 vs. Ctr

To confirm the results obtained using circRNA expression plasmids, we transfected cells with real hsa_circ_0004781 instead of the pcDNA-ZKSCAN-c4781 plasmid. Real hsa_circ_0004781 significantly enhanced the proliferation of AsPC-1 cells (Fig. 9A). In addition, real hsa_circ_0004781 markedly downregulated the expression of miR-9-5p and miR-338-3p and upregulated the mRNA expression of *KLF5* and *ADAM17* (Fig. 9B and C). These results confirm that hsa_circ_0004781 exerts oncogenic effects in PDAC through the miR-9-5p/*KLF5* and miR-338-3p/*ADAM17* axes.

## Discussion

In this study, we identified hsa_circ_00047811 from the GSE79634 and GSE69362 datasets, where it was highly expressed in PDAC tissues. Knockdown of hsa_circ_0004781 significantly inhibited cell proliferation, upregulated miR-9-5p and miR-338-3p expression, and downregulated KLF5 and ADAM17 expression. Conversely, overexpression of hsa_circ_0004781 yielded the opposite results. Moreover, miR-9-5p and miR-338-3p mimics significantly downregulated the protein and mRNA expression of KLF5 and ADAM17, respectively, and inhibited cell proliferation. Conversely, miR-9-5p and miR-338-3p inhibitors upregulated the protein expression of KLF5 and ADAM17, respectively. Finally, real hsa_circ_0004781 was employed to validate the critical results, including increased cell proliferation, downregulation of miR-9-5p and miR-338-3p, and upregulation of KLF55 and ADAM17 expression. These results suggest that hsa_circ_0004781 positively regulate the proliferation of PDAC cells through the miR-9-5p/*KLF5* and miR-338-3p/*ADAM17* axes.

Circinteractome analysis predicted four EIF4A3-binding sites in the upstream and downstream regions of hsa_circ_0004781 pre-mRNA. EIF4A3, an RNA-binding protein, is a crucial regulator of circRNA formation [23, 24]. Therefore, EIF4A3 might bind to the flanking regions of hsa_circ_0004781, facilitating BSJ formation and hsa_circ_0004781 production. Fragile X mental retardation protein (FMRP), another RNA-binding protein, plays a role in regulating mRNA stability and translation [25, 26]. A previous study demonstrated that circZNF609 binds to FMRP, which interacts with RAC1 mRNA to form a circZNF609–FMRP–RAC1 mRNA complex. In this complex, RAC1 mRNA translation is inhibited, and the low level of RAC1 protein limits melanoma metastasis [27]. In the current study, Circinteractome also predicted numerous FMRP-binding sites in hsa_circ_0004781, indicating that hsa_circ_0004781 might inhibit the translation of tumor suppressor genes through interaction with FMRP, thereby promoting cell proliferation and metastasis. However, further experiments are required to confirm this hypothesis.

The results of the present study indicated that hsa_circ_0004781 is highly expressed in PDAC, and it acts as a sponge for miR-9-5p and miR-338-3p. Thus, both miR-9-5p and miR-338-3p are downregulated in PDAC. Previous studies have reported that both miR-9-5p and miR-338-3p are downregulated in PDAC tissues relative to in normal pancreatic tissues [20–22]. However, the mechanisms through which miR-9-5p and miR-338-3p regulate PDAC cell proliferation and migration remain to be elucidated. Our bioinformatics analysis demonstrated that hsa_circ_0004781 may act as a sponge for other miRNAs in addition to miR-9-5p and miR-338-3p (Fig. 4A). However, these miRNAs were not included in the present study because they were not aberrantly expressed in PDAC.

miRNAs typically regulate the expression of many target genes, either positively or negatively. In the present study, we identified five targets each for miR-9-5p and miR-338-3p, and all were noted to be highly expressed in PDAC tissues. Among them, *KLF5* and *ADAM17* were significantly correlated with the poor survival of patients with PDAC. *KLF5* is a transcription factor that activates gene expression by binding to GC-rich promoter regions [28]. *KLF5* is upregulated in PDAC, and its downregulation was reported to inhibit PDAC cell proliferation and tumorigenesis in mice by increasing the expression of the tumor suppressor *NDRG2* and reducing the activation of STAT5 [29]. Previous studies have reported that *KLF5* is a target of miR-9-5p, and the downregulation of miR-9-5p increased the expression of *KLF5*, promoting the proliferation and migration of vascular smooth muscle cells [30] and the proliferation of pulp stem cells [31]. ADAM17, a membrane-anchored protein that exhibits endopeptidase activity, is involved in the activation of the Notch pathway and the maturation of the soluble form of tumor necrosis factor-α [32, 33]. In addition, ADAM17 has an oncogenic function in PDAC [34] and was reported to be required for PDAC development in a mouse genetic study [35]. ADAM17 is upregulated following the knockdown of miR-338-3p, which promotes cancer cell proliferation and metastasis [36–38]. These findings indicate that both *KLF5* and *ADAM17* are crucial molecules for cancer progression, and in the current study, they were noted to be regulated by hsa_circ_0004781 through miR-9-5p and miR-338-3p, respectively. These results suggest that hsa_circ_0004781 plays a key regulatory role in PDAC progression through the miR-9-5p/*KLF5* and miR-338-3p/*ADAM17* axes.

Most studies on relevant topics have used circRNA expression plasmids or viral vectors to indirectly express circRNAs and evaluate their biological functions. These plasmids typically transcribe a linear pre-mRNA containing upstream and downstream Alu elements as well as splice donor and splice acceptor sites derived from the intronic sequences of genes, such as human *ZKSCAN1* and *Drosophila laccase2*. The downstream Alu elements exhibit high complementarity to their upstream counterparts, bringing the splice donor and splice acceptor sites into close proximity and inducing back-splicing to form circRNAs [39, 40]. In our laboratory, we used the pcDNA3.1 ZKSCAN1 MCS Exon vector to express circRNAs. This vector contains multiple restriction enzyme sites for cloning genes of interest between the splice donor and splice acceptor sites of the *ZKSCAN1* gene. However, directly cloning genes into these restriction enzyme sites can result in circRNAs with undesired cloning site sequences. In the present study, we inserted two partial *PCNX1* intronic sequences (approximately 30 nucleotides each) containing SD and SA sites between the SD and SA sites of the *ZKSCAN1* gene, along with exons 2–5 of the *PCNX1* gene. Back-splicing occurred at the SD and SA sites of the *PCNX1* gene instead of the *ZKSCAN1* gene, resulting in the correct formation of hsa_circ_0004781 without undesired cloning site sequences. However, our experience indicates that this strategy is ineffective for most other genes. In most cases, back-splicing occurs at the SD and SA sites of the *ZKSCAN1* gene. Therefore, alternative cloning methods, such as ligation-independent cloning, may be more advantageous for constructing circRNA expression plasmids instead of traditional restriction enzyme/ligase cloning.

circRNA expression plasmids or viral vectors typically generate undesired transcripts, such as unspliced linear pre-mRNA, concatamers, and trans-spliced RNAs [41, 42]. The biological functions of these undesired transcripts might differ from those of circRNA because of variations in sequences and secondary or tertiary structures. Furthermore, the observed biological functions might be attributable to these undesired transcripts instead of the circRNA. Thus, in the present study, we generated real hsa_circ_0004781 by using a group I intron ribozyme to validate key findings obtained using a circRNA expression plasmid. Currently, three types of artificial ribozymes, namely group I intron, group II intron, and hairpin ribozyme, are used to prepare circRNAs [43]. However, most artificial ribozyme permuted intron–exon constructs generate circRNAs with intronic scars that might trigger unwanted innate immune responses [17], introduce unexpected functions, and even disrupt the open reading frame of some translatable circRNAs. Using the group I intron ribozyme, we successfully generated real hsa_circ_0004781 without scar sequences in vitro and confirmed the data obtained using the circRNA expression plasmid in cells. However, our experience indicates that the efficiency of circRNA formation depends on the specificity of the selected target site as well as the sequence and size of the circRNA of interest.

## Conclusions

An increasing number of studies have reported that circRNAs play crucial roles in tumor progression. However, their underlying regulatory mechanisms remain unclear. In the present study, we determined that hsa_circ_0004781 was upregulated in PDAC and promoted PDAC cell proliferation and migration through the miR-9-5p/*KLF5* and miR-338-3p/*ADAM17* axes. Therefore, hsa_circ_0004781 might be a therapeutic target for PDAC.

## Electronic supplementary material

Below is the link to the electronic supplementary material.

Supplementary Material 1

Supplementary Material 2

Supplementary Material 3

Supplementary Material 4

Supplementary Material 5

Supplementary Material 6

Supplementary Material 7

Supplementary Material 8

Supplementary Material 9

Supplementary Material 10

## Data Availability

No datasets were generated or analysed during the current study.

## Abbreviations

ADAM17: A disintegrin and metalloproteinase domain 17
AP2A1: Adaptor related protein complex 2 subunit alpha 1
BLAST: Basic Local Alignment Search Tool
BSJ: Back-splicing junction
circRNA: circular RNA
EMT: Epithelial–mesenchymal transition
FMRP: Fragile X mental retardation protein
HPLC: High-performance liquid chromatography
HMGA2: High-mobility AT-hook 2
IGS: Internal guide sequences
IP-RP: Ion-pair reversed-phase
IVT: In vitro transcription
JAK2: Janus kinase 2
KLF5: Kruppel-like factor 5
KRAS: Kirsten rat sarcoma
miRNA: microRNA
mRNA: messenger RNA
MTT: 3-(4,5-dimethylthiazol-2-yl)-2,5-diphenyltetrazolium bromide
PAGE: Polyacrylamide gel electrophoresis
PCNX1: Pecanex 1
PDAC: Pancreatic ductal adenocarcinoma
pre-mRNA: precursor mRNA
qPCR: quantitative PCR
PTBP1: Polypyrimidine tract-binding protein 1
PTEN: Phosphatase and tensin homolog
RT-PCR: Reverse transcription–polymerase chain reaction
SE: Standard error
SMAD: Mothers against decapentaplegic homolog
SSJ: Self-splicing junction
STAT3: Signal transduction and activator of transcription 3
TGF: Transforming growth factor
YTHDF2: YTH N^6^-methyladenosine RNA-binding protein 2
