# Supplementary figures and images for: CircRNA hsa_circ_0004781 promoted cell proliferation by acting as a sponge for miR-9-5p and miR-338-3p and upregulating KLF5 and ADAM17 expression in pancreatic ductal adenocarcinoma
Source: Cancer Cell Int. 2025 Feb 19;25:56. doi: 10.1186/s12935-025-03687-0 (PMC11841339; doi:10.1186/s12935-025-03687-0)

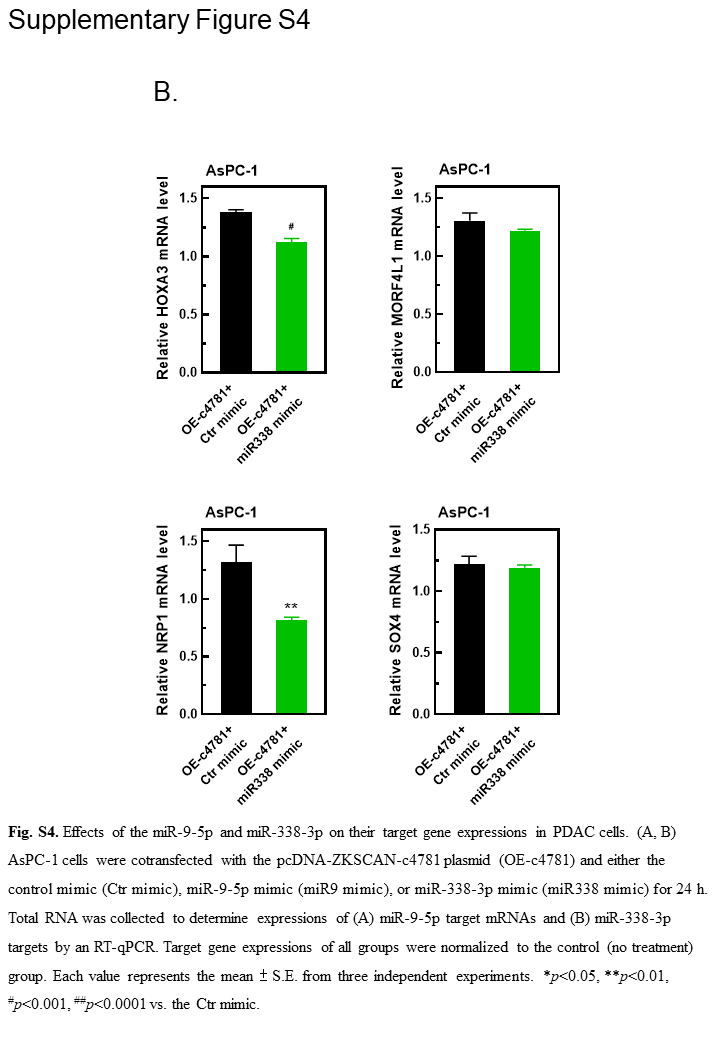

Supplement: Supplementary file 1 — Supplementary Material 1 [file 12935_2025_3687_MOESM1_ESM.tif]

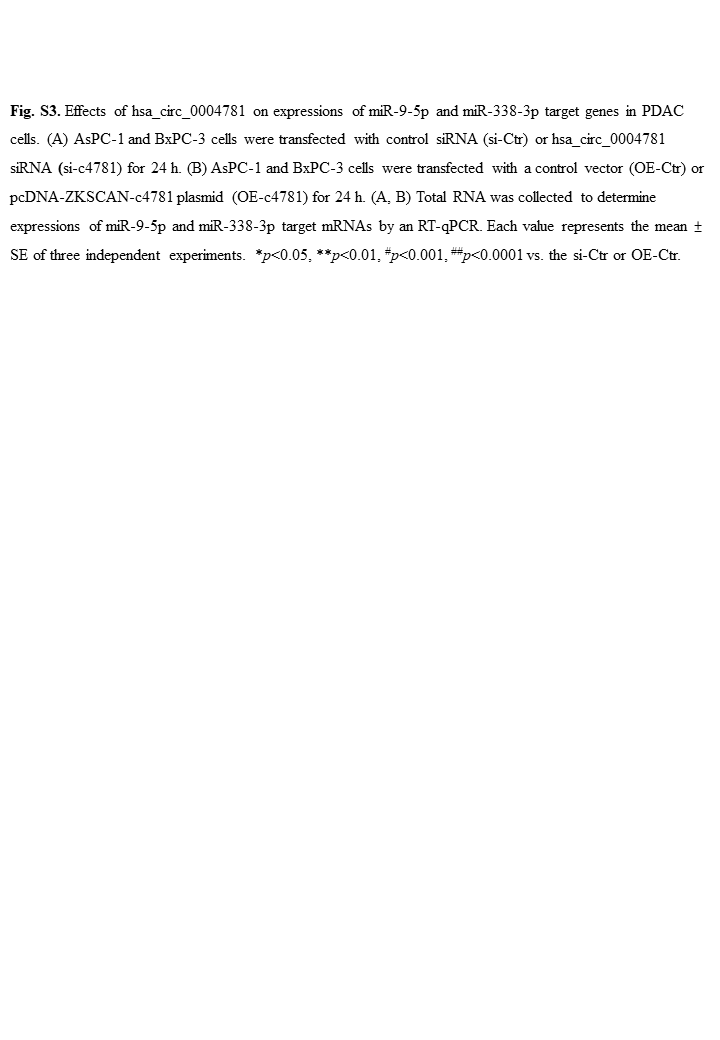

Supplement: Supplementary file 2 — Supplementary Material 2 [file 12935_2025_3687_MOESM2_ESM.tif]

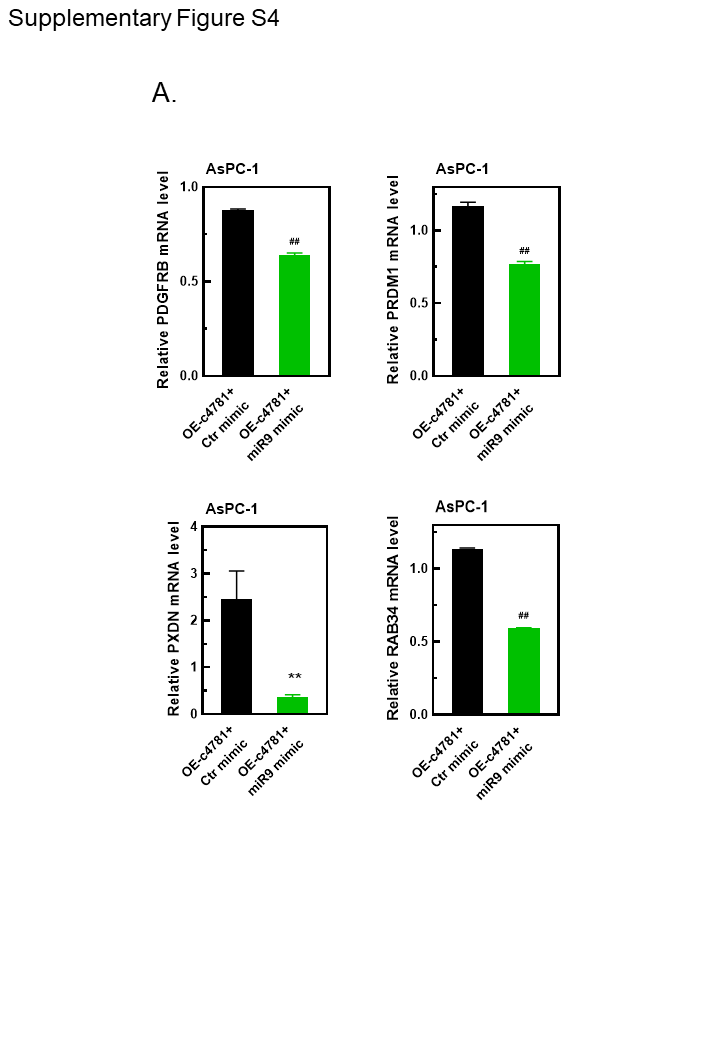

Supplement: Supplementary file 3 — Supplementary Material 3 [file 12935_2025_3687_MOESM3_ESM.tif]

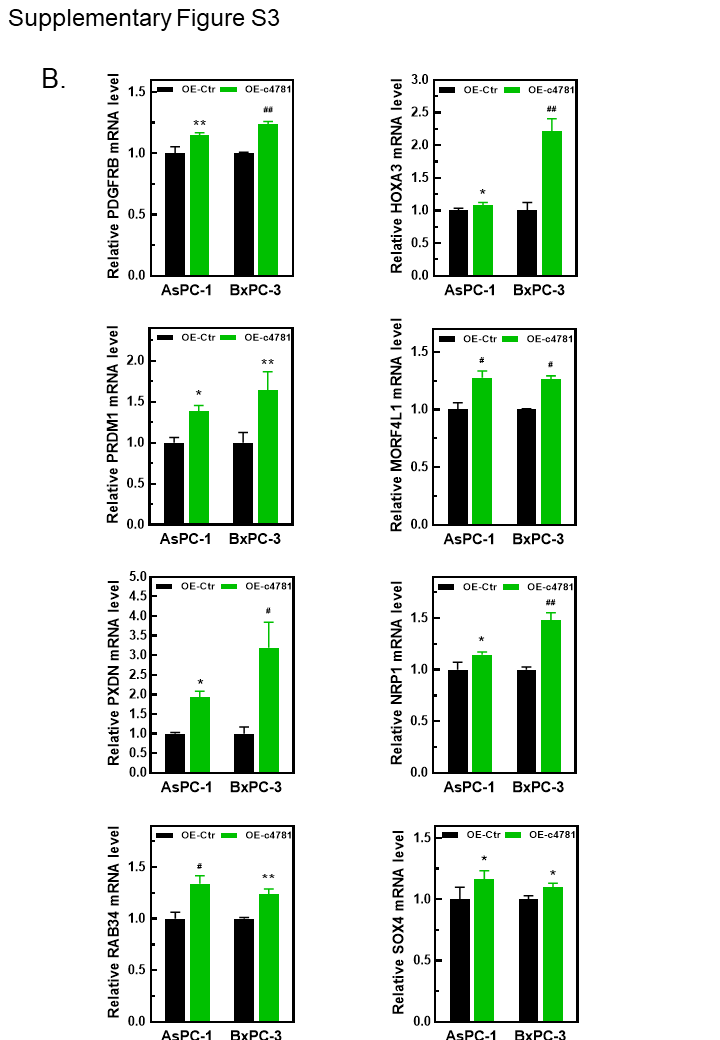

Supplement: Supplementary file 4 — Supplementary Material 4 [file 12935_2025_3687_MOESM4_ESM.tif]

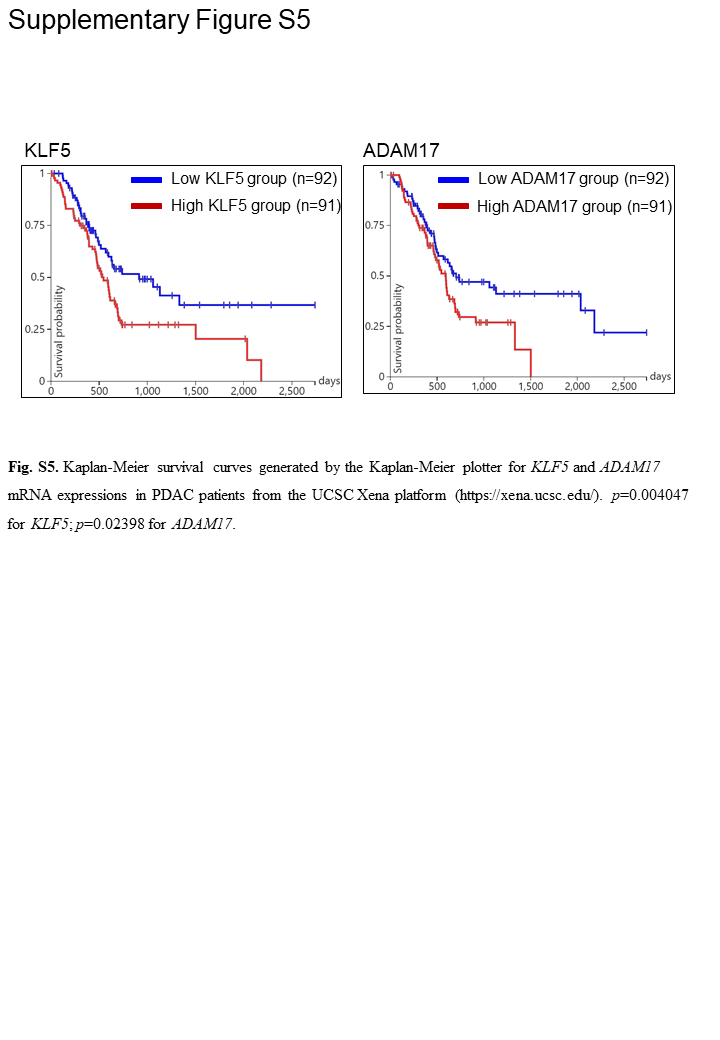

Supplement: Supplementary file 5 — Supplementary Material 5 [file 12935_2025_3687_MOESM5_ESM.tif]

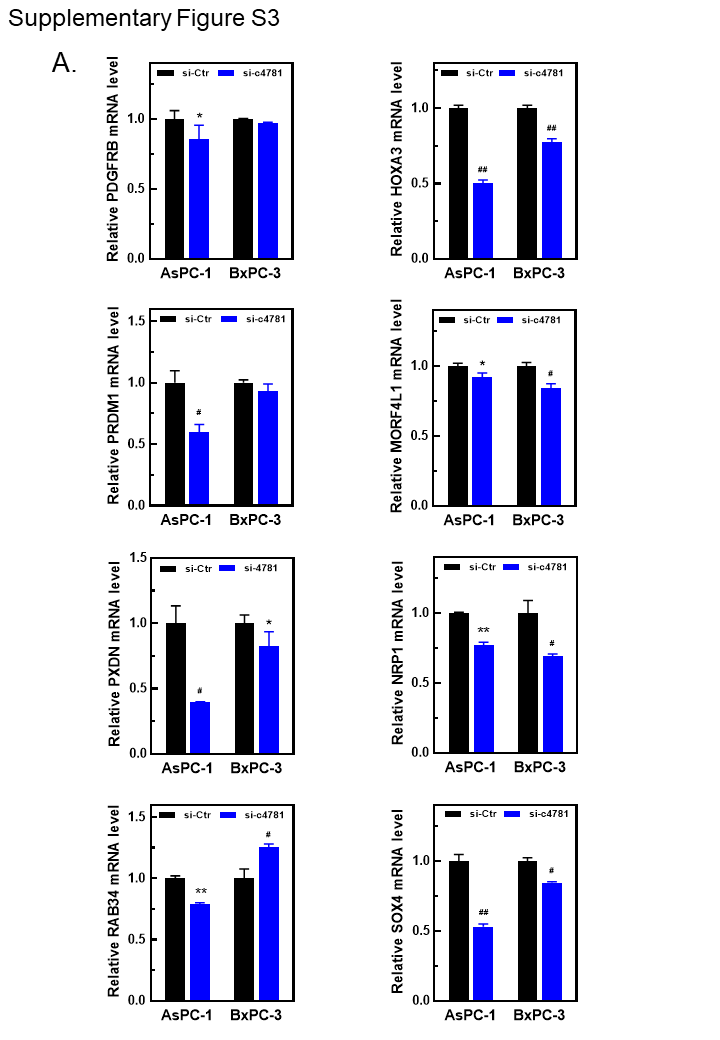

Supplement: Supplementary file 6 — Supplementary Material 6 [file 12935_2025_3687_MOESM6_ESM.tif]

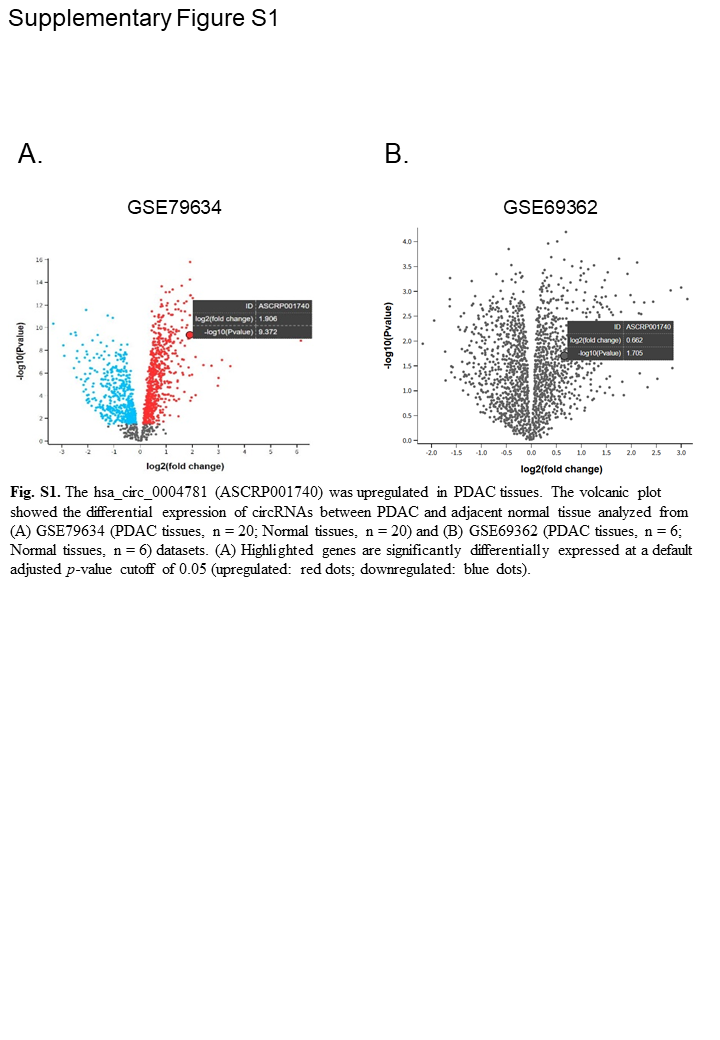

Supplement: Supplementary file 7 — Supplementary Material 7 [file 12935_2025_3687_MOESM7_ESM.tif]

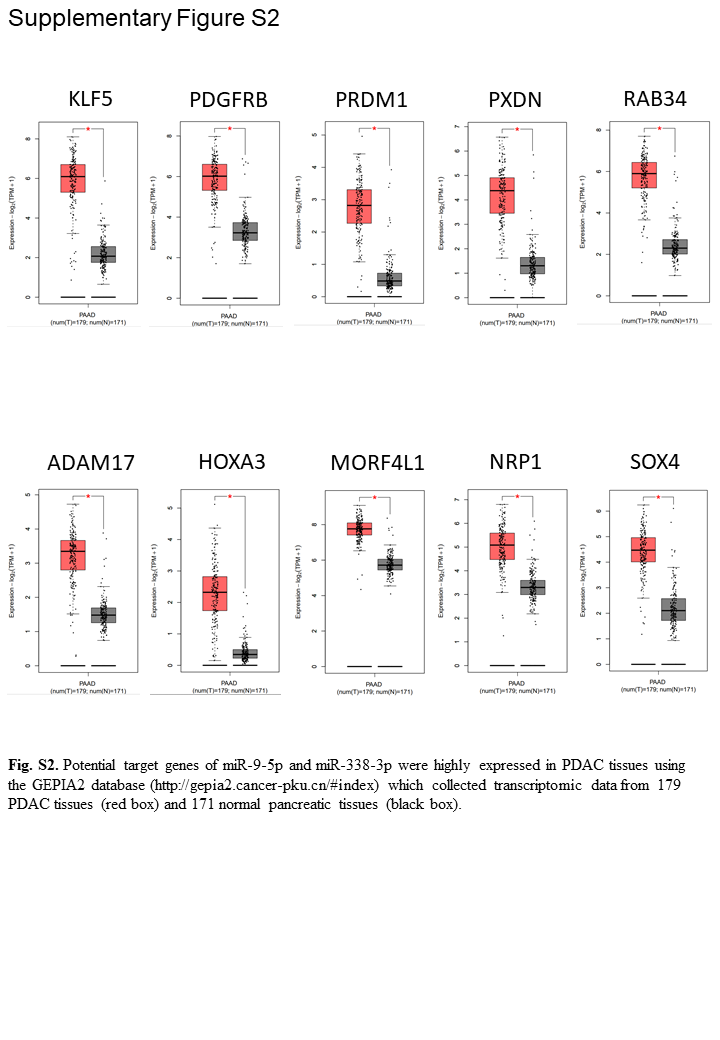

Supplement: Supplementary file 8 — Supplementary Material 8 [file 12935_2025_3687_MOESM8_ESM.tif]
