# Supplementary material for: CircRNA hsa_circ_0004781 promoted cell proliferation by acting as a sponge for miR-9-5p and miR-338-3p and upregulating KLF5 and ADAM17 expression in pancreatic ductal adenocarcinoma
Source: Cancer Cell Int. 2025 Feb 19;25:56. doi: 10.1186/s12935-025-03687-0 (PMC11841339; doi:10.1186/s12935-025-03687-0)
Supplement: Supplementary file 9 — Supplementary Material 9 [file 12935_2025_3687_MOESM9_ESM.docx]

**Supplementary Table S1. The primer sequences used in qPCR and PCR.**

| Primer names | Primer sequences for qPCR |
| --- | --- |
| KFL5 forward | 5’-GGAGAAACGACGCATCCACTAC-3’ |
| KFL5 reverse | 5’-GAACCTCCAGTCGCAGCCTTC-3’ |
| PDGERB forward | 5’-TGCAGACATCGAGTCCTCCAAC-3’ |
| PDGERB reverse | 5’-GCTTAGCACTGGAGACTCGTTG-3’ |
| PRDM1 forward | 5’-CAGTTCCTAAGAACGCCAACAGG-3’ |
| PRDM1 reverse | 5’-GTGCTGGATTCACATAGCGCATC-3’ |
| PXDN forward | 5’-GTCGTGGCCCACCTGACTG-3’ |
| PXDN reverse | 5’-GTGTCGCTGGGAATGCTG-3’ |
| RAB34 forward | 5’-ATGCGCTGATGGAGAAAGACGC-3’ |
| RAB34 reverse | 5’-CTCGGACATTCTCACCAGTGAG-3’ |
| ADAM17 forward | 5’-AACAGCGACTGCACGTTGAAGG-3’ |
| ADAM17 reverse | 5’-CTGTGCAGTAGGACACGCCTTT-3’ |
| HOXA3 forward | 5’-CCTGCTCAACTCACCCACAGTG-3’ |
| HOXA3 reverse | 5’-TCTTGTCGCCAGCGCAGCTTTC-3’ |
| MORF4L1 forward | 5’-ATGGTGGCAGTACCAGTGAGAC-3’ |
| MORF4L1 reverse | 5’-GCCACGGTTTTAGCTCTTCAGG-3’ |
| NRP1 forward | 5’-AACAACGGCTCGGACTGGAAGA-3’ |
| NRP1 reverse | 5’-GGTAGATCCTGATGAATCGCGTG-3’ |
| SOX4 forward | 5’-GACATGCACAACGCCGAGATCT-3’ |
| SOX4 reverse | 5’-GTAGTCAGCCATGTGCTTGAGG-3’ |
| GAPDH forward | 5’-AGCCACATCGCTCAGACAC-3’ |
| GAPDH reverse | 5’-GCCCAATACGACCAAATCC-3’ |
| hsa-miR-9-5p forward | 5’-TCTTTGGTTATCTAGCTGTATGA-3’ |
| hsa-miR-338-3p forward | 5’-TCCAGCATCAGTGATTTTGTTG-3’ |
| Universal miRNA reverse | 5’-GAACATGTCTGCGTATCTC-3’ |
| Primer names | Primer sequences for PCR |
| c4781 divergent forward | 5’-TCTGGGCCAAAGTTCTAGTCT-3’ |
| c4781 divergent reverse | 5’-TGATCCGTGAACTCATTTGC-3’ |
| c4781 convergent 1 forward | 5’-ACAGAGCACTTGATGCTGGA-3’ |
| c4781 convergent 1 reverse | 5’-CTCCTGGTTGAACAGTTGCC-3’ |
| c4781 convergent 2 forward | 5’-TTCCTCGCGTCTTGGAACAG-3’ |
| c4781 convergent 2 reverse | 5’-GGAGTCATTGGAGACAAGACGA-3’ |

c4781, hsa_circ_0004781.
