# Supplementary material for: CircRNA hsa_circ_0004781 promoted cell proliferation by acting as a sponge for miR-9-5p and miR-338-3p and upregulating KLF5 and ADAM17 expression in pancreatic ductal adenocarcinoma
Source: Cancer Cell Int. 2025 Feb 19;25:56. doi: 10.1186/s12935-025-03687-0 (PMC11841339; doi:10.1186/s12935-025-03687-0)
Supplement: Supplementary file 10 — Supplementary Material 10 [file 12935_2025_3687_MOESM10_ESM.docx]

**Supplementary Table S2. Sequences of the siRNAs, miRNA mimics and miRNA inhibitors used in the present study.**

c4781, hsa_circ_0004781.

| Sequences names | Sequences |
| --- | --- |
| siRNA for c4781 | 5′-GUGAAGAACAAGGCCCUUCCUATdT-3′ |
|  | 3′-dTdTCACUUCUUGUUCCGGGAAGGA-5′ |
| hsa-miR-9-5p mimic | 5′-UCCAGCAUCAGUGAUUUUGUUG-3′ |
|  | 3′-AUACAGCUAGAUAACCAAAGAUU-5′ |
| hsa-miR-338-3p mimic | 5′-UCUUUGGU-U AUCUAGCUGUAUGA-3′ |
|  | 3′-ACAAAAUCACUGAUGCUGGAUU-5′ |
| control miRNA mimic | 5′-UUCUCCGAACGUGUCACGUTT-3′ |
|  | 3′-ACGUGACACGUUCGGAGAATT-5′ |
| hsa-miR-9-5p inhibitor | 5′-UCAUACAGCUAGAUAACCAAAGA-3′ |
| hsa-miR-338-3p inhibitor | 5’-CAACAAAAUCACUGAUGCUGGA-3’ |
| control miRNA inhibitor | 5′-CAGUACUUUUGUGUACAA-3′ |
